# Homozygous expression of the myofibrillar myopathy-associated p.W2710X filamin C variant reveals major pathomechanisms of sarcomeric lesion formation

**DOI:** 10.1186/s40478-020-01001-9

**Published:** 2020-09-04

**Authors:** Julia Schuld, Zacharias Orfanos, Frédéric Chevessier, Britta Eggers, Lorena Heil, Julian Uszkoreit, Andreas Unger, Gregor Kirfel, Peter F. M. van der Ven, Katrin Marcus, Wolfgang A. Linke, Christoph S. Clemen, Rolf Schröder, Dieter O. Fürst

**Affiliations:** 1 Institute for Cell Biology, Department of Molecular Cell Biology, University of Bonn, Ulrich-Haberland-Str. 61a, 53121 Bonn, Germany; 2 Present address: Institute of Virology, University Hospital Giessen and Marburg, Marburg, Germany; 3 Institute of Neuropathology, University Hospital Erlangen, Friedrich-Alexander University Erlangen-Nürnberg, Schwabachanlage 6, 91054 Erlangen, Germany; 4 present address: CureVac AG, Tübingen, Germany; 5 Medizinisches Proteom-Center, Ruhr-University Bochum, Bochum, Germany; 6 Institute of Physiology II, University of Münster, Münster, Germany; 7 Center for Biochemistry, Institute of Biochemistry I, Medical Faculty, University of Cologne, Cologne, Germany; 8 Center for Physiology and Pathophysiology, Institute of Vegetative Physiology, Medical Faculty, University of Cologne, Cologne, Germany; 9 Institute of Aerospace Medicine, German Aerospace Center (DLR), Cologne, Germany

**Keywords:** Myofibrillar myopathy, Mouse model, Filamin, Myofibrillar lesions, Muscle damage, BAG3, HSPB7, Pathophysiology, Autophagy

## Abstract

Filamin C (FLNc) is mainly expressed in striated muscle cells where it localizes to Z-discs, myotendinous junctions and intercalated discs. Recent studies have revealed numerous mutations in the *FLNC* gene causing familial and sporadic myopathies and cardiomyopathies with marked clinical variability. The most frequent myopathic mutation, p.W2710X, which is associated with myofibrillar myopathy, deletes the carboxy-terminal 16 amino acids from FLNc and abolishes the dimerization property of Ig-like domain 24. We previously characterized “knock-in” mice heterozygous for this mutation (p.W2711X), and have now investigated homozygous mice using protein and mRNA expression analyses, mass spectrometry, and extensive immunolocalization and ultrastructural studies. Although the latter mice display a relatively mild myopathy under normal conditions, our analyses identified major mechanisms causing the pathophysiology of this disease: in comparison to wildtype animals (i) the expression level of FLNc protein is drastically reduced; (ii) mutant FLNc is relocalized from Z-discs to particularly mechanically strained parts of muscle cells, i.e. myotendinous junctions and myofibrillar lesions; (iii) the number of lesions is greatly increased and these lesions lack Bcl2-associated athanogene 3 (BAG3) protein; (iv) the expression of heat shock protein beta-7 (HSPB7) is almost completely abolished. These findings indicate grave disturbances of BAG3-dependent and -independent autophagy pathways that are required for efficient lesion repair. In addition, our studies reveal general mechanisms of lesion formation and demonstrate that defective FLNc dimerization via its carboxy-terminal domain does not disturb assembly and basic function of myofibrils. An alternative, more amino-terminally located dimerization site might compensate for that loss. Since filamins function as stress sensors, our data further substantiate that FLNc is important for mechanosensing in the context of Z-disc stabilization and maintenance.

## Introduction

Heterozygous mutations in the human filamin C gene (*FLNC*) located on chromosome 7q32 cause hereditary and sporadic myopathies and cardiomyopathies with marked phenotypic variability [18, 23, 32, 86]. The first disease-causing *FLNC* mutations were described in the context of skeletal myopathies displaying the classical features of myofibrillar myopathies (MFMs; MIM# 609524), which are morphologically characterized by sarcoplasmic protein aggregates and degenerative changes of the myofibrillar apparatus [23, 67, 69, 86]. In addition, *FLNC* mutations were subsequently demonstrated to cause distal myopathies (MIM# 614065) without evidence for protein aggregation [16, 26]. More recently, a growing number of truncating and missense *FLNC* mutations have been identified in cardiac diseases comprising restrictive, hypertrophic, dilated and arrhythmogenic cardiomyopathies (MIM# 617047) [4, 5, 9, 18, 25, 56, 78]. A very recent extensive review provides a complete overview of the currently known approximately 325 *FLNC* variants. A large part of those are associated with skeletal myopathies and cardiomyopathies [83].

Filamin C (FLNc) is a large, dimeric, actin-cross-linking protein with a molecular mass of approximately 2 × 290 kDa. It is predominantly expressed in cross-striated muscle cells, and localizes to myofibrillar Z-discs, myotendinous junctions (MTJs), intercalated discs and the sarcolemma [80]. Its modular molecular architecture, consisting of an actin-binding domain followed by 24 immunoglobulin-like (Ig-like) domains, allows for multiple simultaneous protein-protein interactions, making FLNc a multi-adapter protein. Accordingly, more than 90 proteins have been identified as FLNc ligands, emphasizing its involvement in multiple scaffolding and signaling functions in muscle cells [52, 79, 91]. The essential role of FLNc for muscle development and maintenance is further highlighted by the effect of the abolition of its expression in mice and zebrafish. Loss of FLNc in mice causes a severe, perinatal lethal phenotype including defects in embryonic myogenesis resulting in a decreased number of primary fibers, excessive fiber size variation, and disturbance of sarcomere architecture [14]. In another mouse model, depletion of FLNc in the embryonic heart resulted in fetal death, while loss of FLNc in the hearts of adult mice caused severe dilated cardiomyopathy in most mice, leading to early death [92]. Corresponding studies in zebrafish demonstrated myofibril failure and altered sarcomeric architecture, but no effects on primary myogenesis [64].

In the present study, we explore the pathophysiology of the human *FLNC* p.W2710X mutation [ClinVar: NM_001458.4(*FLNC*):c.8130G > A (p.Trp2710Ter); Allele ID: 33353], which in the heterozygous state is the most frequently encountered human myopathy-causing *FLNC* gene defect in various ethnic groups [37, 86]. It must be noted that another mutation mainly appearing in the population of Hong Kong [ClinVar: NM_001458.4(*FLNC*):c.8129G > A (p.Trp2710Ter); Allele ID: 682327] leads to the expression of an identical protein variant [44]. Both truncation mutations result in loss of the 16 carboxy-terminal amino acids, thus impeding the formation of dimers of Ig-like domain 24 of FLNc. As a result, the mutant protein has a strong tendency to aggregate and undergo proteolytic degradation [47, 86]. Our previously reported heterozygous p.W2711X knock-in mice, harboring the murine orthologue of the human *FLNC* p.W2710X mutation, represent the first patient-mimicking genetic animal model for human filaminopathies [11]. These mice primarily demonstrated that heterozygous expression of this pathogenic FLNc variant results in myofibrillar instability with formation of FLNc- and Xin-positive sarcomeric lesions [11]. To explore the pathomechanism of this mutation in skeletal muscle more precisely, we now investigated mice homozygous for the *FLNC* p.W2711X mutation (Hom mice). These animals are viable and fertile, demonstrating that the exclusive expression of at least this particular mutant FLNc variant does not interfere with primary myogenesis. However, in comparison to wildtype (WT) and heterozygous animals [11], total FLNc levels were markedly reduced and concomitantly the protein was strikingly redistributed to areas of particular mechanic strain in skeletal muscle fibers. Moreover, these mice showed increased abundance of Z-disc streaming exemplified by the presence of sarcomeric microlesions (spanning up to five sarcomeres) and macrolesions (more than five sarcomeres and across multiple myofibrils), thus highlighting that FLNc might have a pivotal role for the structural and functional integrity of the myofibrillar apparatus. The dramatic decrease in the expression level of the autophagy-related small heat-shock protein HSPB7 in the muscle fibers of our Hom mice, and the absence of BAG3 from myofibrillar lesions in these mice suggest a cooperative role of these proteins in protection against, and repair of sarcomeric damage.

## Materials and methods

### The *Flnc* p.W2711X knock-in mouse model

The *Flnc* c.8133G > A; p.W2711X knock-in mouse model B6J.B6-*Flnc*^tm1.1Rsdf^ (http://www.informatics.jax.org/allele/MGI:5907163) was generated as described before [11]. In brief, a point mutation in exon 48 of the *Flnc* gene leading to the insertion of a stop codon instead of a tryptophan residue was inserted by homologous recombination (GenOway, Lyon, France). The mutation was extensively validated by different methods. Genotyping was performed as described [11].

Mice were housed in isolated ventilated cages (IVC) equipped with spruce granulate embedding and a nest under specific and opportunistic pathogen-free (SOPF) conditions at 22 ± 2 °C, 50 to 70% air humidity, 70 air exchanges per h, and a light-dark-cycle of 12/12 h with free access to water and food. Littermates were separated at weaning by sex and housed at maximal five animals per cage. Health monitoring was done as recommended by the Federation of European Laboratory Animal Science Associations (FELASA). Mice were handled in accordance with the German Animal Welfare Act and the German Regulation for the protection of animals used for experimental purposes or other scientific purposes. All investigations were approved by the governmental office for animal care (reference number 84–02.05.40.14.057).

### Histology, immunochemistry and light microscopy

Soleus muscles from WT and mutant mice (*n* = 3–4 per genotype) were snap frozen in liquid nitrogen cooled isopentane and stored at − 80 °C. To assess histology, 5 μm thick transverse cryosections were stained with haematoxylin and eosin (HE) and modified Gömöri trichrome, and for cytochrome oxidase (COX) and succinate dehydrogenase (SDH) activity according to standard procedures.

For immunostaining, 10 μm thick transverse or 6 μm thick longitudinal cryosections were fixed with acetone (− 20 °C) for 10 min or for 2 min with methanol (− 20 °C) followed by 30 s acetone (− 20 °C), respectively. After fixation, sections were rehydrated with phosphate-buffered saline (PBS) and incubated with blocking medium (10% normal goat serum in PBS) for 45 min at 37 °C. Sections were incubated with primary antibodies diluted in 1% bovine serum albumin (BSA) in PBS overnight at 4 °C. After washing with PBS containing 0.05% Tween 20 (PBST), sections were incubated at 37 °C with the appropriate secondary antibodies diluted in 1% BSA in PBS for at least 1 h. Slides were rinsed in PBS and dipped in water before being mounted in Mowiol containing 0.25% n-propyl gallate (Sigma P3130). Images were acquired using an LSM710 confocal laser scanning or a Cell Observer SD spinning disc microscope (Carl Zeiss GmbH, Oberkochen, Germany).

### Infrared western blot analysis and protein quantification

For comparative protein quantification, muscle samples from 6 to 10-month-old WT and mutant mice were dissected (*n* = 4 per genotype), weighed and snap frozen in liquid nitrogen. Samples were mechanically disrupted using a TissueLyser LT (Qiagen, Hilden, Germany) at 50 Hz and dissolved in 15 μl urea buffer [2 M thiourea, 7 M urea, 5 mM EDTA, 1 mM DTT and protease inhibitors (Sigma, P8340) in 100 mM Tris pH 8.6] per 1 mg of muscle sample by homogenization for 3 min at 50 Hz. Preheated SDS sample buffer was added to a final concentration of 2-fold and samples were incubated for 5 min at 55 °C. After quantitative analysis of a Coomassie-stained SDS-polyacrylamide gel, total protein concentration of the lysates was adjusted. For comparative quantitative blotting, identical total protein amounts were separated by SDS-polyacrylamide gel electrophoresis (SDS-PAGE) as previously described [42] and transferred onto a polyvinylidenfluoride (PVDF) membrane using a Transblot SD blot apparatus (BioRad, Munich, Germany). Membranes were incubated with Odyssey blocking buffer (LI-COR Biosciences, Bad Homburg, Germany) for 30 min at room temperature and incubated overnight at 4 °C with primary antibodies diluted in Tris-buffered saline with 0.05% Tween 20 (TBST). Subsequently, membranes were washed in TBST and incubated with IRDye-680 or IRDye-800-conjugated secondary antibodies (LI-COR Biosciences, Bad Homburg, Germany). Samples were analyzed using a LI-COR Odyssey Infrared Imaging System (LI-COR Biosciences). Integrated intensities of protein bands were quantified using the Odyssey Infrared Imaging Software v. 3.0 (LI-COR Biosciences) and normalized to α-tubulin or glyceraldehyde-3-phosphate dehydrogenase (GAPDH).

### Antibodies

The following antibodies were used in this study: anti-FLNc RR90, recognizing FLNa and FLNc [80], the rabbit sera FLNc d16–20 labelling Ig-like domains 16–20, and FLNc WT recognizing the carboxy-terminal 16 amino acids of Ig-like domain 24 of WT FLNc [11] were previously described. The mouse monoclonal antibodies (mAbs) XR1, labelling the isoforms XinA and XinB [82], T12 which recognizes a titin epitope close to the Z-disc [22], BB78 against myomesin [84], the rat mAb YL1/2 against tyrosinated α-tubulin [88] and the rabbit serum detecting sarcomeric α-actinin (RaA653) [80] have also been described previously. Antibodies recognizing type I, IIA IIX and IIB myosin heavy chain (MHC) isoforms (BA-F8, SC71, 6H1, BF-F3, respectively) and anti-dystrophin (MANDRA1) were obtained from Developmental Studies Hybridoma Bank **(**DSHB, Iowa City, USA). Anti-GAPDH (clone 5C6) was purchased from Calbiochem/Merck Millipore (Darmstadt, Germany), anti-vinculin (hVin-1, V9131) from Sigma Aldrich/Merck, (Darmstadt, Germany), anti-slow MHC (NCL-MHCs) from Novocastra/Leica Microsystems (Wetzlar, Germany), and anti-BAG3 and anti-HSPB7 from Proteintech, Manchester, UK. Secondary antibodies conjugated to Texas Red, Alexa Fluor 488, 594, 647, Cy2, Cy3 and Cy5 were from SouthernBiotech (Birmingham, AL, USA) or from Jackson ImmunoResearch (Ely, UK). For more detailed information on antibodies see Additional file 1: Table S1.

### RNA extraction and real-time quantitative PCR

To analyze *Flna*, *Flnb* and *Flnc* expression levels, RNA was isolated from the soleus muscles of 8-month-old WT and Hom animals using RNeasy Fibrous Tissue Mini Kit (Qiagen, Hilden, Germany) in combination with TissueLyser LT (Qiagen, Hilden, Germany). Genomic DNA was eliminated by RNase-free DNase I treatment. One microgram of total RNA was used for cDNA synthesis by the Omniscript RT Kit (Qiagen, Hilden, Germany) using random hexamer primers, according to the recommendations of the manufacturer. Real-time quantitative PCR reactions were performed using the SsoFast EvaGreen Supermix (BioRad, Munich, Germany) in a total reaction volume of 15 μl and the CFX96 real-time PCR detection system (BioRad, Munich, Germany). For quantification, all reactions were performed in triplicates from 3 to 6 different cDNA samples prepared from different mice. The expression levels obtained were normalized to that of *Gapdh* and β2 microglobulin (*B2m*). To determine the relative levels of mRNA expression between Hom and WT samples, ratios were calculated by the ΔΔCT method [46] using the Biorad CFX manager software version 3.1 and included a correction for amplification efficiency of the individual transcripts. Sequences of the applied oligonucleotides are provided in Additional file 1: Table S2.

### Electron microscopy

Soleus muscle samples from 8-month-old WT and mutant mice (*n* = 2 per genotype) were immersed in Karnovsky fixative [33] containing 2.5% glutaraldehyde, 2% paraformaldehyde in 0.1 M cacodylate buffer, pH 7.4 for 2 d at 4 °C. After washing twice with 0.1 M cacodylate buffer, samples were trimmed to a smaller size and post-fixed in 1% OsO_4_ for 1 h at room temperature. Samples were rinsed three times with double distilled water and counterstained with 4% uranyl acetate. Dehydration was carried out in a graded series of ethanol (50, 70, 95% and twice in 100% ethanol) for 15 min each followed by 3 consecutive steps of propylene oxide (Merck, Darmstadt, Germany). Samples were embedded in Epon resin (Serva, Heidelberg, Germany) which was allowed to polymerize for 48 h at 60 °C. To identify the specimens containing micro- and/or macrolesions, 1 μm thick semithin sections were stained with toluidine blue and photographed (Axio Imager M1, Carl Zeiss, Jena, Germany). From these blocks 70 nm ultrathin sections were prepared (Ultracut S; Leica, Germany) that were placed on 2 × 1 mm copper slot grids (Plano, Wetzlar, Germany) and examined with a Verios 460 L microscope (FEI - Thermo Fisher Scientific, Eindhoven, the Netherlands).

### Protein interaction and crosslinking assays

A human *FLNC* cDNA fragment comprising Ig-like domains 15–23 was amplified with a full-length *FLNC* construct as template, and cloned into modified pAct2 and pLex vectors using standard protocols. Similarly, truncated variants of this fragment were cloned into the pLex vector. Transformation into L40 yeast cells, and culturing and testing for β-galactosidase activity was performed as described previously [81].

Protein cross-linking assays were performed essentially as described [29]. In brief, His_6_-tagged recombinant proteins were expressed in *E.coli*, purified using Ni-NTA agarose, and dialyzed against cross-link buffer (CLB; 600 mM NaCl, 50 mM NaP_i_, pH 7.4, 1 mM MgCl_2_, and 1 mM DTT). Subsequently, 50 μl of a 10 μM protein solution in CLB was equilibrated at 37 °C for 10 min, and 0.5 μl of 65 mM ethylene glycol bis (succinimidyl succinate) (EGS) in DMSO, or DMSO alone as a control, was added. The mixture was incubated for a further 10 min at 37 °C. After the reaction was stopped by addition of SDS sample buffer and incubation for 10 min at 95 °C, 5 μl were analyzed by SDS-PAGE and western blotting using an antibody against the immunotag of the recombinant proteins.

Glutathione S-transferase (GST) pulldown assays were performed as described [51] using GST-tagged FLNc d18–21, and His_6_- and T7-tagged FLNc d18–21 and FLNc d15–17, respectively.

### Laser microdissection, sample processing and mass spectrometry

The identification of different fiber types before laser microdissection (LMD) was enabled by staining for anti-slow MHC as described [89]. Non-fluorescent fibers were classified as type II fibers. A total of 250,000 μm^2^ of the sarcoplasmic area of fibers was excised, collected and further treated for LMD as described [89], except that samples were reduced in 6.7 mM dithiothreitol and alkylated with 5 mM iodoacetamide. Approximately 200 ng peptides (16 μl) of each sample was used for label-free quantitative mass spectrometric (MS) analyses, performed on an UltiMate 3000 RSLC nano LC system (Dionex, Idstein, Germany) as described [49]. After each sample the column was equilibrated by a 1 h washing step. The HPLC system was online-coupled to the nano ESI source of a Q Exactive mass spectrometer (Thermo Fisher Scientific, Germany). In the ESI-MS/MS analysis, full MS spectra and MS/MS scans were performed as described [10] with the exception that only ions with a charge of + 2 and + 3 were selected for fragmentation.

### Data analysis of mass spectrometry

Raw files were converted into mgf format using Proteome Discoverer 1.4 (Thermo Fisher Scientific, Germany) and then searched against a combined database containing the Swiss-Prot part of UniProt KB [76] for *Mus musculus* (version 2018_06, 16,985 entries) and the common Repository of Adventitious Proteins as contaminant database (version 2012.01.01, 115 entries). Shuffled decoy entries were generated for each protein using DecoyDatabaseBuilder [60]. For the identification of spectra, a respective concatenated target-decoy-database was used. Identifications were performed using Mascot 2.5 [Matrixscience Ltd., [59]], allowing 2 missed cleavages, precursor tolerance of 5 ppm and fragment tolerance of 20 mmu. Carbamidomethylation (Cys) was set as static and carbamidomethylation (Asn), deamidation (Asn, Gln) and oxidation (Met) as variable modification. A spectral counting workflow was created in KNIME [version 3.5.3, [6]] to allow automated processing of sample analysis. The mass spectrometry proteomics data have been deposited to the ProteomeXchange Consortium via the PRIDE [58] partner repository with the dataset identifier PXD020097. Mascot dat files were used as starting point. First, the samples were processed by PIA [73, 77] to receive the peptide spectrum matches (PSMs) needed for spectral counting. The false discovery rate (FDR) was calculated for all files separately and an FDR of 1% on PSM level was maintained. The resulting PSMs were filtered for proteotypicity, i.e. only peptides, which were assigned to exactly one protein in the given database, were kept for further analyses. Here, the assignment to a contaminant was not considered to break the proteotypicity. The number of PSMs for each protein per run were normalized on all spectra per run, while also the non-proteotypic identifications were counted. These normalized counts are called spectral indices for each protein.

In order to ensure comparability of the data with previous experiments on myofibrillar myopathy patients, statistical analysis was performed as described [39, 48]. In brief: to identify significantly differentially expressed proteins, the ratio of the average spectral indices between phenotypes were calculated and Student’s t-test was conducted on the spectral indices for each protein. Proteins with a *p*-value < 0.05 were considered as significantly differentially expressed between the different phenotypes.

### General data analysis and figure preparation

Data analysis and statistical evaluations were performed using GraphPad Prism version 7 for Windows (GraphPad Software, La Jolla, CA, USA) or Microsoft Excel 2016 in conjunction with the add-in “Real Statistics Resource Pack” version 5.5 by Charles Zaiontz, available at http://www.real-statistics.com (version 3.2.3). Data are given as median with IQR or as mean ± SEM. *P*-values (Hom vs. WT) were calculated by Student’s t-test, Kruskal-Wallis one-way analysis of variance and Mann–Whitney U (Wilcoxon rank-sum) test and quoted * < 0.05, ** < 0.01, *** < 0.001 and **** < 0.0001 (precise choice of test given in Figure legends). Final assembly of the graphs and preparation of all figures was done using Corel Draw Graphics Suite X7 (Corel, Austin, TX, USA).

## Results

### Filamin C is expressed at high levels in the soleus muscle of mice

**Fig. 1:**
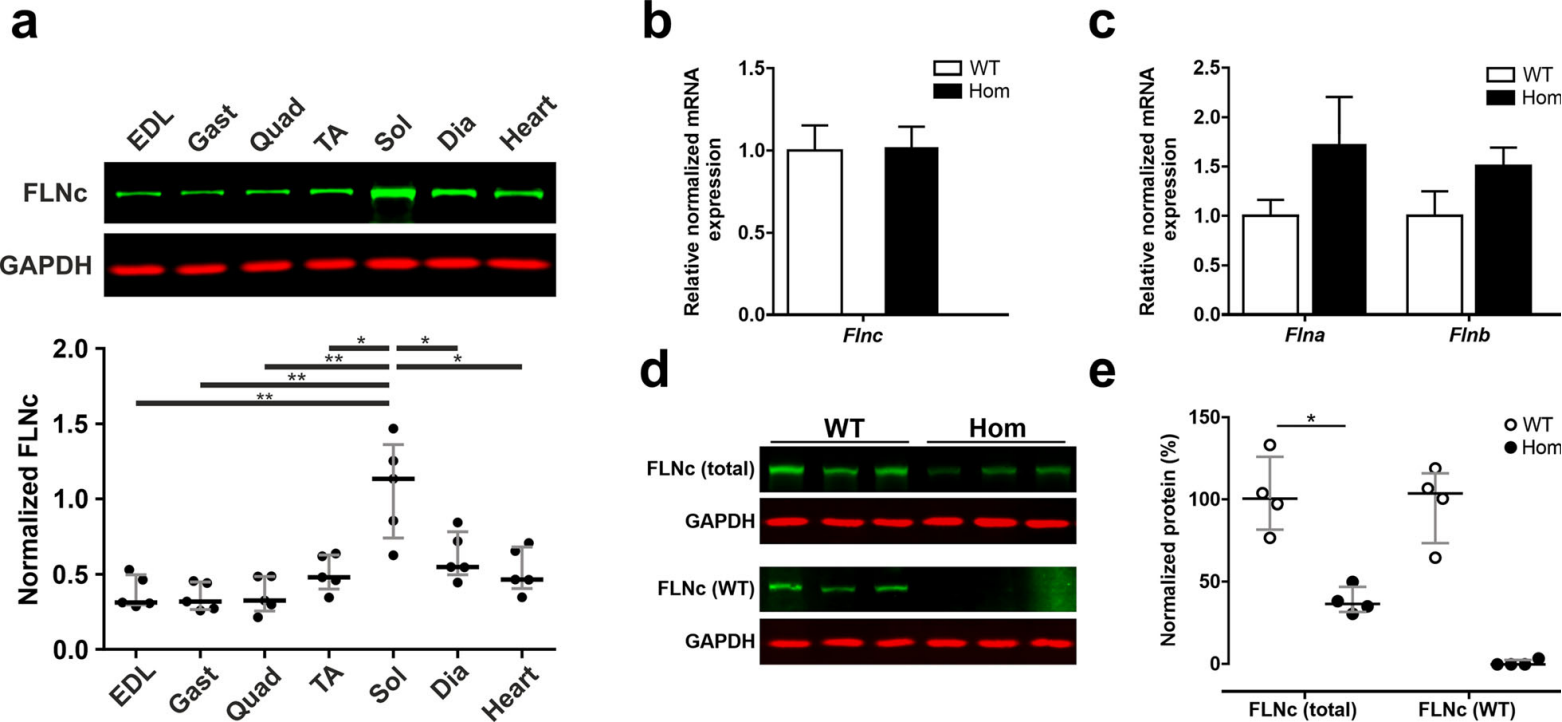
FLNc expression in different mouse muscles, and soleus muscle of wildtype and Hom mice. **a** Protein quantification in different wildtype mouse muscles demonstrated highest FLNc expression levels in soleus muscle. Anti-GAPDH was used as loading and normalization control. *n* = 5. EDL: Musculus (M.) extensor digitorum longus; Gast: M. gastrocnemius; Quad: *M. quadriceps*; TA: *M. tibialis* anterior; Sol: M. soleus; Dia: diaphragm. **b** Quantitative real time PCR showed virtually identical total *Flnc* mRNA levels in adult WT and Hom animals (*n* = 4–6). **c** Comparison of *Flna* and *Flnb* mRNA expressions using the Biorad CFX manager software version 3.1 and Student’s t-test revealed no significant differences in Hom animals (n = 4–6). **d**, **e** Western blotting of total soleus muscle extracts from adult mice using an antibody recognizing both, WT and mutant FLNc (FLNc total) in conjunction with protein quantification using a LI-COR Odyssey Infrared Imaging System. Statistical analysis using Mann-Whitney U-testing indicated a significant reduction to approximately 40% of total FLNc expression in Hom animals. Results are shown as mean +/− IQR (n = 4; *p* = 0.0286). Staining with an antiserum recognizing the carboxy-terminal 16 amino acids of FLNc (and thus only WT FLNc) confirmed its complete absence in Hom animals (*n* = 3)

To determine which striated muscles express the highest FLNc levels, protein extracts from different skeletal muscles and the heart from WT mice were analyzed by western blotting. Quantification using the LI-COR Odyssey Infrared Imaging System demonstrated that the expression level of FLNc in the soleus muscle is significantly higher than in all other skeletal muscles, the diaphragm or the heart (Fig. 1a). For this reason, we decided to analyze the effects of the expression of only mutant FLNc in soleus muscle.

### Comparison of the expression levels of filamin C mRNA and protein in WT and Hom mice

Reverse transcription quantitative polymerase chain reaction (RT-qPCR) to analyze *Flnc* mRNA expression revealed no significant differences between total *Flnc* mRNA levels in the soleus muscles of WT and Hom animals (Fig. 1b). Similarly, expression levels of *Flna* and *Flnb* mRNA were analyzed. Their expression levels were not significantly increased in soleus muscles of Hom mice (Fig. 1c).

**Fig. 2:**
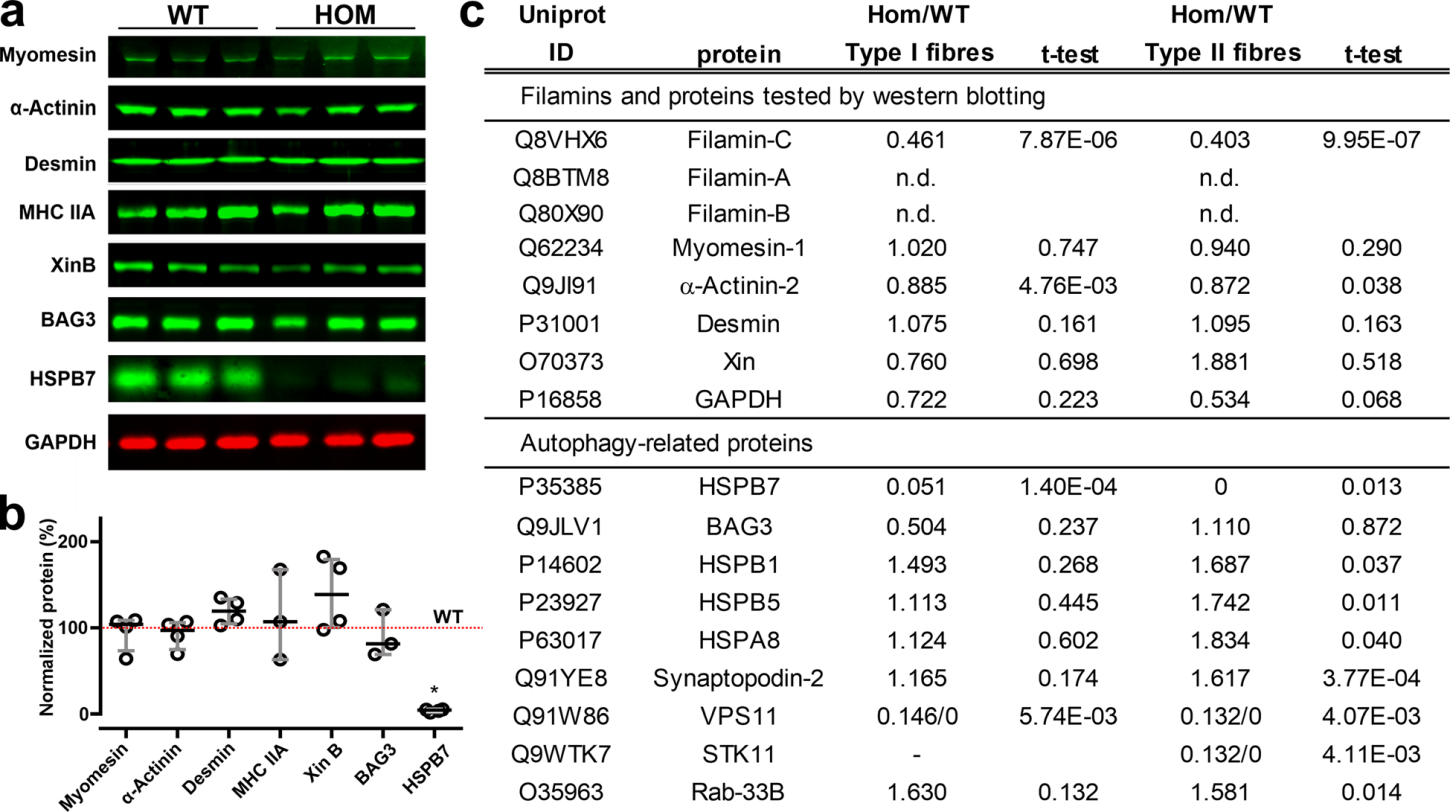
Expression of sarcomeric and autophagy-related proteins in wildtype and Hom soleus muscle. **a** Western blotting revealed no differences in expression of the M-band component myomesin, the Z-disc component α-actinin2, intermediate filaments (desmin), the thick filament component MHC IIa, the muscle damage marker Xin and the molecular chaperone regulator BAG3, whereas the small heat-shock protein HSPB7 showed a striking reduction in Hom mice. GAPDH was used as loading control. **b** Quantification of the results shown in panel A revealed no significant differences in the expression levels of all analyzed proteins, except for the almost complete absence of HSPB7 in Hom mice (n = 4; p = 0.0286; Mann-Whitney U-test). **c** Proteomic analysis of laser dissected sarcoplasma of soleus muscle fibers from Hom and WT mice confirmed that the proportion of FLNc in Hom mice was reduced to 46 and 40% in type I and type II fibers, respectively, whereas FLNa and FLNb were not detectable. Investigation of other proteins tested by western blotting, revealed a slight underrepresentation of α-actinin2, whereas myomesin, desmin and Xin levels were not changed. Note that MHC IIa is not yet represented in the UniProt KB database [76], and could therefore not be detected in the mass spectrometric analyses. The autophagy related protein HSPB7 was hardly detectable in both fiber types of Hom mice, whereas other small heat shock proteins such as HSPB1 and HSPB5 are significantly upregulated in type II fibers. Notably, other autophagy-related proteins, such as synaptopodin-2, VPS11, STK11 and RAB-33B also show a significantly increased expression in type II fibers of our Hom mice (Student’s t-test)

We then analyzed the FLNc protein levels in our mice by western blotting, using two different antisera: one, directed against Ig-like d16–20, recognizes all FLNc, whereas the second one is directed against the 16 carboxy-terminal amino acids absent in the mutant and only reacts with the WT protein. This revealed that the total FLNc level in Hom mice was reduced to approximately 40% of that in WT mice (Fig. 1d, e). Staining with the second antiserum gave no signal in the Hom mice, confirming the complete absence of WT FLNc and the exclusive expression of mutant FLNc in those animals (Fig. 1d, e). We further confirmed this by proteomic analysis of the FLNc expression level in different fiber types of the soleus muscle of Hom and WT animals. In accordance with the data obtained by western blotting, in type I and type II fibers FLNc levels were reduced to 46.1 and 40.3% of the corresponding levels in WT muscle fibers, respectively (Fig. 2c).

Comparison of expression levels of proteins representing different sarcomeric sub-assemblies by quantitative western blotting did not reveal any significant differences in M-band, Z-disc, intermediate filament and A-band proteins or the muscle damage marker Xin (Fig. 2a, b) between WT and Hom mice. These data were grossly confirmed by proteomic analysis (Fig. 2c). By contrast, expression levels of the small heat shock protein HSPB7 (cvHsp, Hsp25–2) were hardly detectable in the muscle fibers of our Hom animals. Again, our western blot data were confirmed by proteomic analysis (Fig. 2a-c). Notably, the latter method, based on MS analysis did not detect a single HSPB7 peptide in 90% of the samples acquired from type I or type II fibers derived from Hom mice. In addition, proteomic analysis indicated that FLNa and FLNb are neither expressed in the sarcoplasm of type I, nor of type II fibers (Fig. 2c). Protein levels of the chaperone-assisted selective autophagy (CASA)-related SYNPO2 (synaptopodin-2) protein were significantly increased in type II fibers of Hom mice, whereas type I fibers also showed a slight, but non-significant increase. Several other autophagy-related proteins were also increased in especially type II fibers (Fig. 2c).

### Homozygosity for the *Flnc* p.W2711X mutation causes a myopathic phenotype

**Fig. 3:**
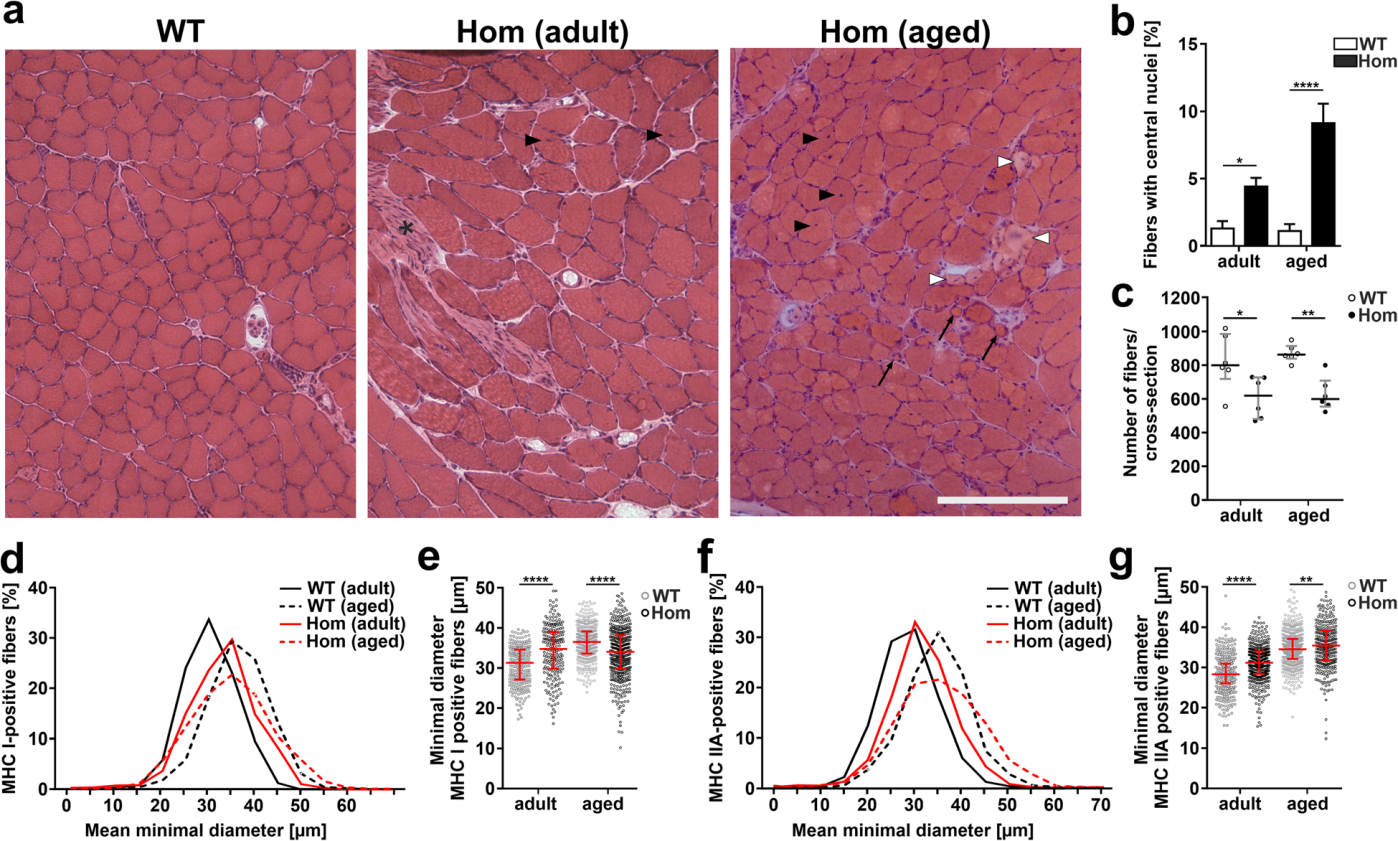
Histological staining and analysis of fiber size, fiber number and fiber type in M. soleus. **a** Hematoxylin and eosin-stained transverse cryosections of WT and Hom mice. The sections of the Hom mice show increased fibrosis (asterisk), several fibers with central nuclei (black arrowheads), and very small fibers (arrows) and degenerating fibers in the aged Hom mice (white arrowheads), indicating an increased myopathic phenotype in these animals. **b** Statistical evaluation of respective percentages of fibers with centrally located nuclei using Kruskal-Wallis test and chi-squared independence tests. The number of muscle fibers with centrally located nuclei is significantly increased in adult and aged Hom animals (adult: *n* = 6; *p* = 0.0033; aged: *n* = 6, *p* < 0.0001). **c** The total number of fibers is significantly lower in the soleus muscle of Hom animals (adult: *n* = 6; *p* = 0.0152; aged: *n* = 6, *p* = 0.0317). **d**-**e** When compared to WT mice, the mean minimal diameter of type I fibers is significantly larger in adult Hom animals (****p < 0.0001), whereas they are smaller in aged mice (****p < 0.0001), indicating that in Hom mice type I fibers don’t increase their size as much as in WT animals during aging. **f**-**g** When compared to WT mice, the mean minimal diameter of type II fibers is significantly larger in adult and aged Hom animals (****p < 0.0001, **p = 0.0061)

In transverse cryosections from soleus muscles of adult (8–10 months) and aged (20 months) WT and Hom mice (4 mice per genotype) stained with haematoxylin and eosin (HE; Fig. 3a), we investigated the number of fibers with centrally located nuclei. Numbers were statistically evaluated using a chi-squared test and demonstrated an increase in the number of centrally located nuclei in adult Hom animals (Hom: 4.40 ± 0.52 vs WT: 1.28 ± 0.44, *n* = 3, *p* = 0.0033) and even more in aged Hom animals (Hom: 9.13 ± 1.16, *n* = 4 vs WT: 1.1 ± 0.40, *n* = 3, *p* < 0.0001, Fig. 3b; Kruskal-Wallis test, Chi-square). Soleus muscles of Hom animals showed a slight increase of endomysial connective tissue, a rounding of muscle fibers and multiple atrophic muscle fibers. In addition, Hom soleus muscles contained degenerating fibers, which were never observed in WT muscles (Fig. 3a). Moreover, in nearly all soleus muscle samples the conspicuous presence of areas with increased fibrosis sometimes resembling myotendinous junctions was noted (Fig. 3a, asterisk). Staining with modified Gömöri trichrome, SDH and COX showed no evidence of mitochondrial abnormalities in Hom animals (Additional file 1: Figure S1).

To investigate the effect of mutant FLNc expression on fiber type and fiber size distribution in Hom animals, cryosections of soleus muscles were analyzed for expression of fiber type specific MHC, followed by statistical evaluation (Supp. Figure S2A-C). This revealed that fiber type composition is not significantly different in the soleus muscles of Hom mice compared to WT mice (Additional file 1: Figure S2B,C). Moreover, fiber number and fiber size (minimal diameter) were analyzed in cryosections stained for dystrophin. Adult and aged Hom soleus muscles contained significantly fewer fibers (Mann-Whitney U-test, 6 mice per genotype; adult: WT: median 795 fibers, IQR 266 vs Hom: 614 fibers, IQR 245, *p* = 0.0152; aged: WT: median 858 fibers, IQR 75 vs Hom: 596 fibers, IQR 155, *p* = 0.0043) (Fig. 3c), although soleus muscle weight compared to body weight was not decreased. This lack of fibers was compensated by a significantly increased fiber size for MHC I-positive fibers in adult Hom mice (Kruskal-Wallis with post-hoc analysis using the Mann-Whitney U-test) 3 mice per genotype; WT: 31.3 μm; 275 fibers, IQR 7.4 vs Hom: 34.8 μm; 216 fibers, IQR 9.2, *p* < 0.0001) as well as MHC IIA-positive fibers in both, adult and aged Hom mice (3 mice per genotype; adult: WT: 28.3 μm; 623 fibers, IQR 5.0 vs Hom: 31.4 μm; 422 fibers, IQR 6.0; 3 mice per genotype, *p* < 0.0001; aged: WT: 34.7 μm; 560 fibers IQR 5.0 vs Hom: 35.6 μm; 326 fibers, IQR 7.6;, *p* = 0.0061) (Fig. 3d-g). By contrast, the size of MHC I-positive fibers in aged mice (3 mice per genotype), was significantly reduced in Hom mice: WT: 36.5 μm; 407 fibers, IQR 5.5 vs Hom: 34.1 μm; 452 fibers, IQR 8.4; *p* < 0.0001. (Fig. 3d-g).

### Reduced expression and altered distribution of mutant filamin C in Hom mice

**Fig. 4:**
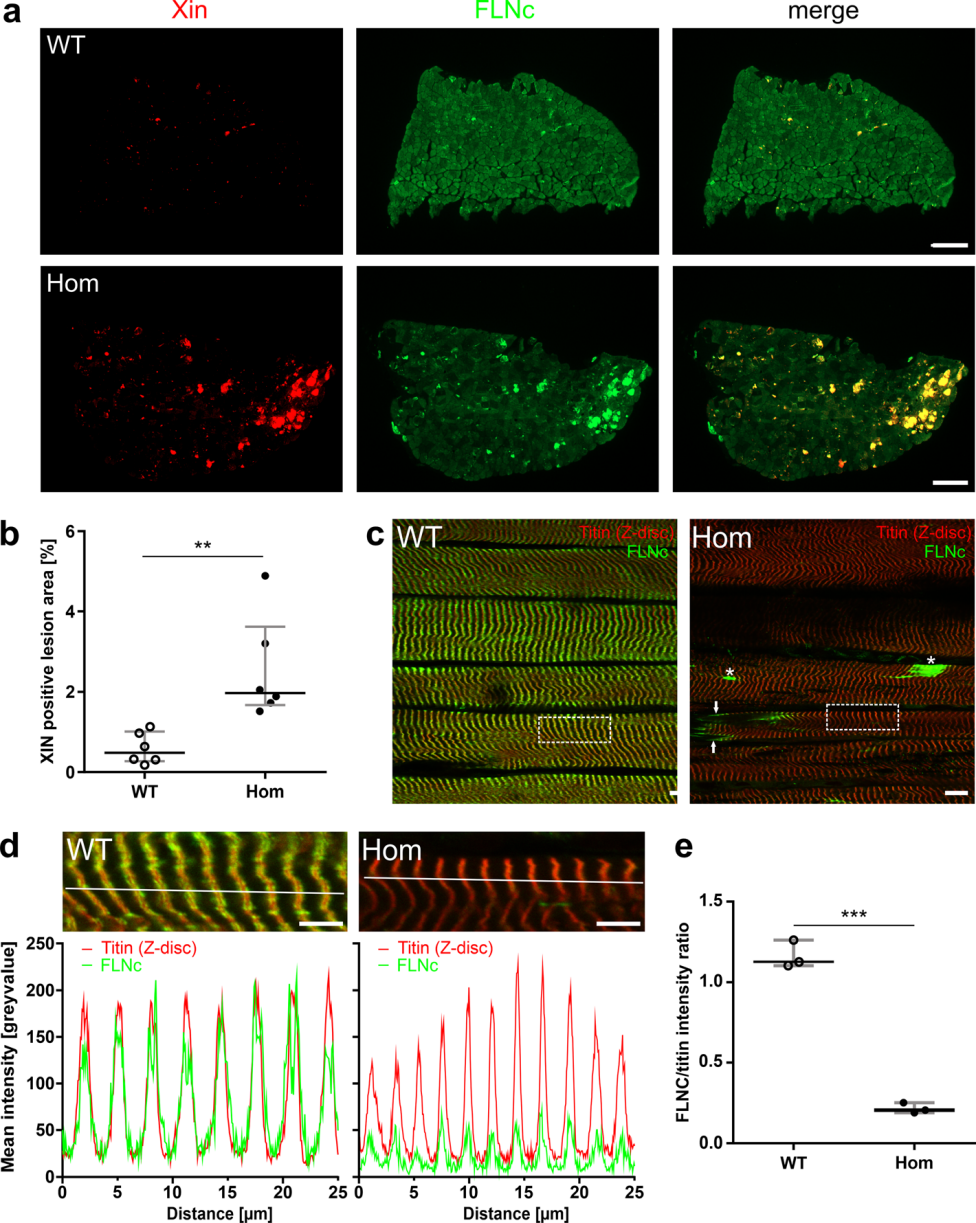
Muscle damage and FLNc content in soleus muscles of adult WT and Hom mice. **a** Immunostaining of soleus muscle cross sections for muscle damage marker Xin reveals increased muscle damage in Hom animals. FLNc colocalizes with Xin in damage areas. Note that identical scan settings were applied. **b** Statistical analysis of the area of muscle damage using the Mann-Whitney U-test reveals significantly increased damage in Hom mice: median of lesion area in % of total muscle area, 0.48 in WT versus 1.97 (n = 6 in each group; *p* = 0.0022). **c**-**e** Comparison of FLNc and titin expression levels in soleus muscle of wildtype and Hom mice. **c** Double staining of longitudinal sections of soleus muscle for FLNc and an epitope of titin close to the Z-disc shows significantly reduced levels of FLNc in Z-discs of Hom mice. Note that identical scan settings were used. Instead, FLNc was found highly concentrated in lesions (asterisks) and MTJs (arrows). **d** Illustration of Zen software line profiles of the mean immunofluorescent intensities of FLNc and titin in Z-discs of WT and Hom mice using the line profile option of the microscope and Zen 2 software Version 2.0.0.0. Note comparable intensities of FLNc and titin signals in Z-discs of wildtype mice but reduced FLNc intensity in Hom mice. **e** Quantification of FLNc Z-disc signal corrected against the background and normalized against titin confirms a highly significant reduction of FLNc levels in Z-discs in Hom mice. Statistical analysis was performed for sections from 3 mice of each genotype and in each 10 Z-discs were included in the calculations. Kruskal-Wallis test one-way analysis of variance; post-hoc analyses by performing Mann-Whitney U-tests of both groups (median of WT: 1.16, median of Hom: 0.22; *p* < 0.0001). For all experiments the RR90 antibody was used to detect FLNc. Bars: 200 μm (**a**), 10 μm (**c**), 5 μm (**d**)

Cross sections of soleus muscles were analyzed for localization of FLNc and the muscle damage marker Xin. Whereas in the muscle fibers of WT animals FLNc was homogeneously expressed in all muscle fibers, soleus muscles from Hom animals only showed minimal staining (Fig. 4a). Instead, brightly stained foci were observed that also contained Xin, indicating that these structures represented muscle damage areas. Analysis of the Xin-positive area compared to the complete area of muscle fibers indicated that Hom fibers displayed significantly more muscle damage (median of lesion area in % of WT: 0.48 vs Hom: 1.97; *p* = 0.0022; 6 mice per genotype; Mann-Whitney U-test Fig. 4b). Staining of longitudinal sections unequivocally identified these intensely stained structures as sarcomeric micro- and macrolesions, rather than amorphous aggregates (Fig. 4c). Whereas in WT soleus muscles large quantities of FLNc were localized to Z-discs, in Hom muscle fibers staining of Z-discs was far less intense. Instead, FLNc was detected mainly in lesions and MTJs (arrowheads in Fig. 4c; Additional file 1: Figure S3). The signal intensity of FLNc localized to these structures was compared to that of titin using the Zen software line profile tool of the confocal microscope (Fig. 4d) and by comparison of the mean FLNc grey value normalized to the titin grey value in individual Z-discs (Fig. 4e). Statistical analysis using Kruskal-Wallis- and subsequent Mann-Whitney U-test revealed a highly significant reduction of signal intensities of FLNc in the Z-discs of Hom soleus muscle fibers (Fig. 4e).

### Distribution of titin and filamin C in macrolesions

**Fig. 5:**
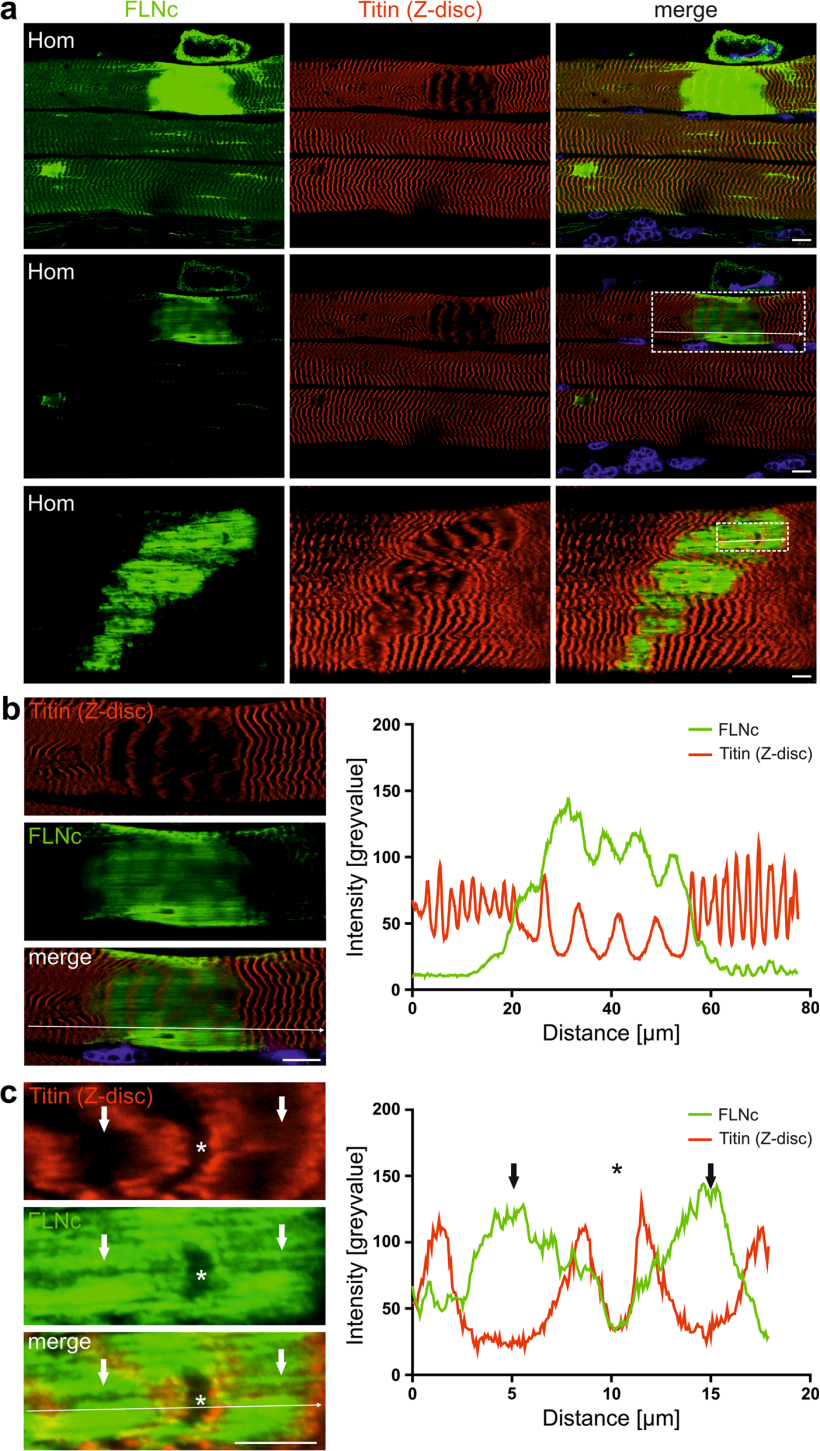
Distribution of FLNc and titin in myofibrillar lesions. **a** Longitudinal cryosections of soleus muscle from an adult Hom mouse stained for FLNc and titin. The upper panel shows an image with increased gain settings necessary to reveal localization of mutant FLNc in Z-discs. Macro- and microlesions are strongly stained. The middle panel shows the identical part of the section with a gain setting enabling localization of FLNc within lesions. The lower panel shows a second fiber with a macrolesion. Boxed areas are shown enlarged in panels **b** and **c**. Note the highly extended distance between Z-discs within lesions, and the localization of FLNc between Z-discs. **b** Line profile of the boxed area in **a** (middle panel) confirms increased signal intensity of FLNc in filamentous appearing structures between Z-discs, while FLNc signals are decreased exactly at the position of the Z-discs. The distance between individual Z-discs increased from 2 to 2.5 μm in normal myofibrils to approximately 7.2 μm (mean distance: 7.21 ± 0.73 μm) in lesions. **c** Line profile of the boxed area in **a** (lower panel). Note a single normal appearing sarcomere within the macrolesion (asterisk) and the absence of FLNc in this area, whereas the space between the titin signals within the lesion is completely filled with FLNc (arrows). RR90 antibody was used to detect FLNc in **a** (lower panel), **b** and **c**. Polyclonal rabbit antiserum against FLNc d16–20 was used in **a** (upper panels). Bars: 10 μm (**a**), 10 μm (**b**), 5 μm (**c**)

Previously, we identified FLNc and Xin as major components of micro- and macrolesions, that are supposed to protect myofibrils against further damage [43, 55]. Analysis of our adult Hom animals revealed high levels of FLNc in these lesions (Fig. 4a, b, Fig. 5a) that showed non-homogeneous FLNc signal intensities (Fig. 5b, c). Co-staining for a Z-disc epitope of titin (Figure 5B,C) combined with a line profile showed that areas with reduced FLNc signals precisely overlap with Z-discs. The distance between Z-disc titin signals within the lesions was approximately 7.2 μm, while in normal appearing sarcomeres the spacing was 2–2.5 μm.

The increased staining for FLNc in MTJs of Hom animals (Fig. 4c), prompted us to directly compare signal intensities of FLNc in these structures. Double staining with an antibody against vinculin to identify MTJs, showed a more intense staining in Hom mice, indicating strong accumulation of mutant FLNc in the MTJs of these mice (Additional file 1: Figure S3).

### Sarcomeric pathology at the ultrastructural level

**Fig. 6:**
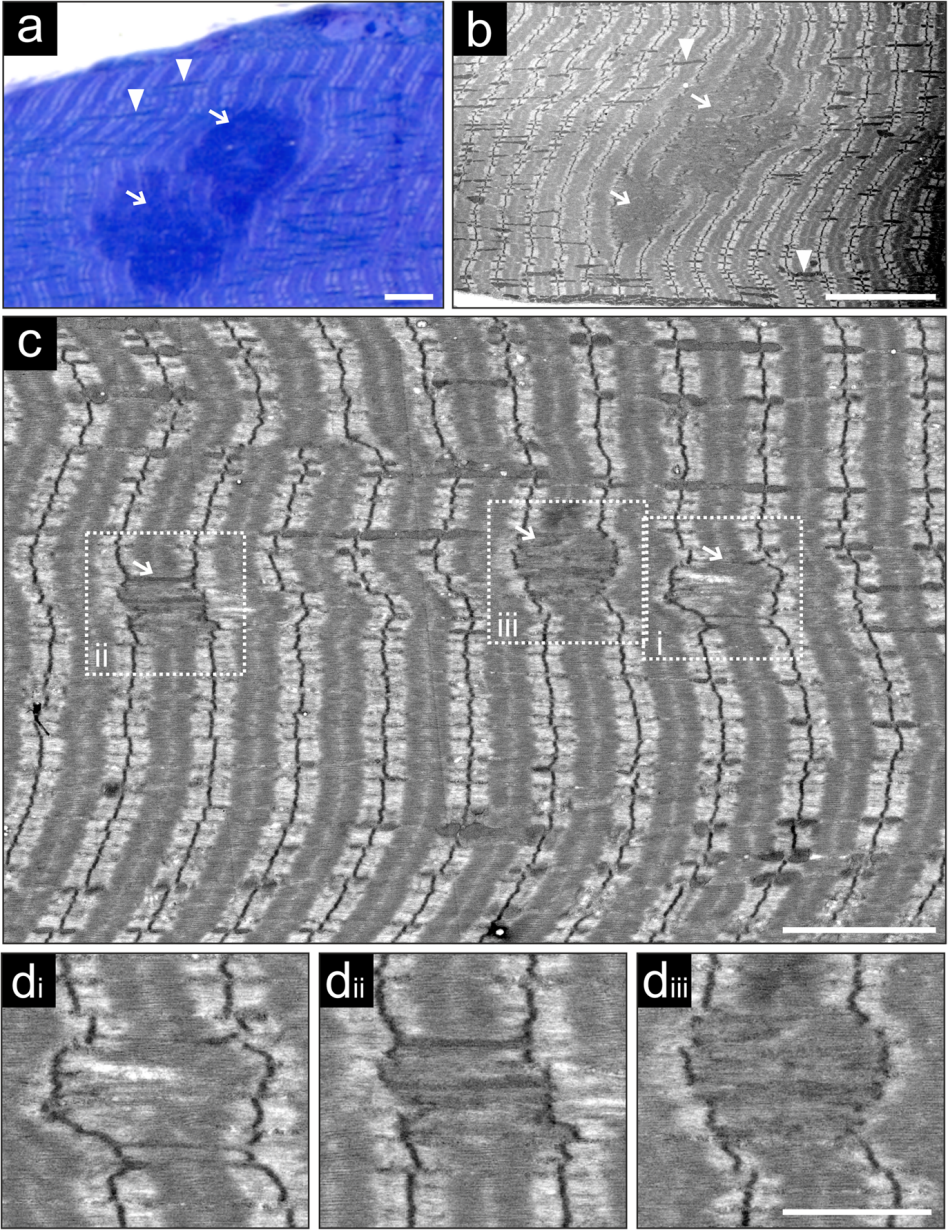
Soleus muscle of Hom mice at the ultrastructural level. **a** Longitudinal semithin section of Epon embedded soleus muscle of an adult Hom mouse stained with toluidine blue. Shown is a section with two neighboring darkly stained macrolesions (arrows). Note numerous elongated and swollen mitochondria (arrowheads). **b** Transmission electron microscopy of an ultrathin section similar to the section shown in A. Depicted are two macrolesions (arrows) surrounded by normally appearing myofibrils. Also, here many elongated and swollen mitochondria are observed (arrowheads). **c** A section containing three microlesions (arrows). The boxed microlesions are shown enlarged in panels **d**_i_-**d**_iii_. Bars: 5 μm (**a**, **c**), 10 μm (**b**), 2.5 μm (**d**_i_-**d**_iii_)

Light microscopy of toluidine blue stained semi thin sections from Epon embedded soleus muscle of adult Hom mice revealed extended dark appearing regions lacking the typical cross striated pattern of the surrounding myofibrils (Fig. 6a). Ultrathin sections of specimens containing such regions with a perfect longitudinal orientation were analyzed by transmission electron microscopy. This confirmed the presence of sarcomeric macrolesions, and showed that they always start at one Z-disc and end at another Z-disc (Fig. 6b). Within macrolesions the normal sarcomere layout of I-bands, A-bands, Z-discs and M-bands was abolished and the space between flanking Z-discs was filled with partially filamentous electron dense material. Notably, these areas were also devoid of mitochondria (Fig. 6b). In addition to macrolesions, many microlesions were observed (Fig. 6c-d), resembling the lesion pathology observed in MFM patient samples. Most microlesions typically extended over a single sarcomere that seemed to gradually lose the normal organization of thin and thick filaments (Fig. 6di), and displayed increasingly disorganized filamentous material. Simultaneously, the distance between the flanking Z-discs increased (Fig. 6dii-diii). Furthermore, we frequently observed rows of elongated and swollen intermyofibrillar mitochondria (Fig. 6a-c) as well as subsarcolemmal autophagic vacuoles (not shown) as previously described for heterozygous p.W2711X animals [11].

### Analysis of BAG3 and HSPB7 localization in muscle fibers from WT and Hom mice

**Fig. 7:**
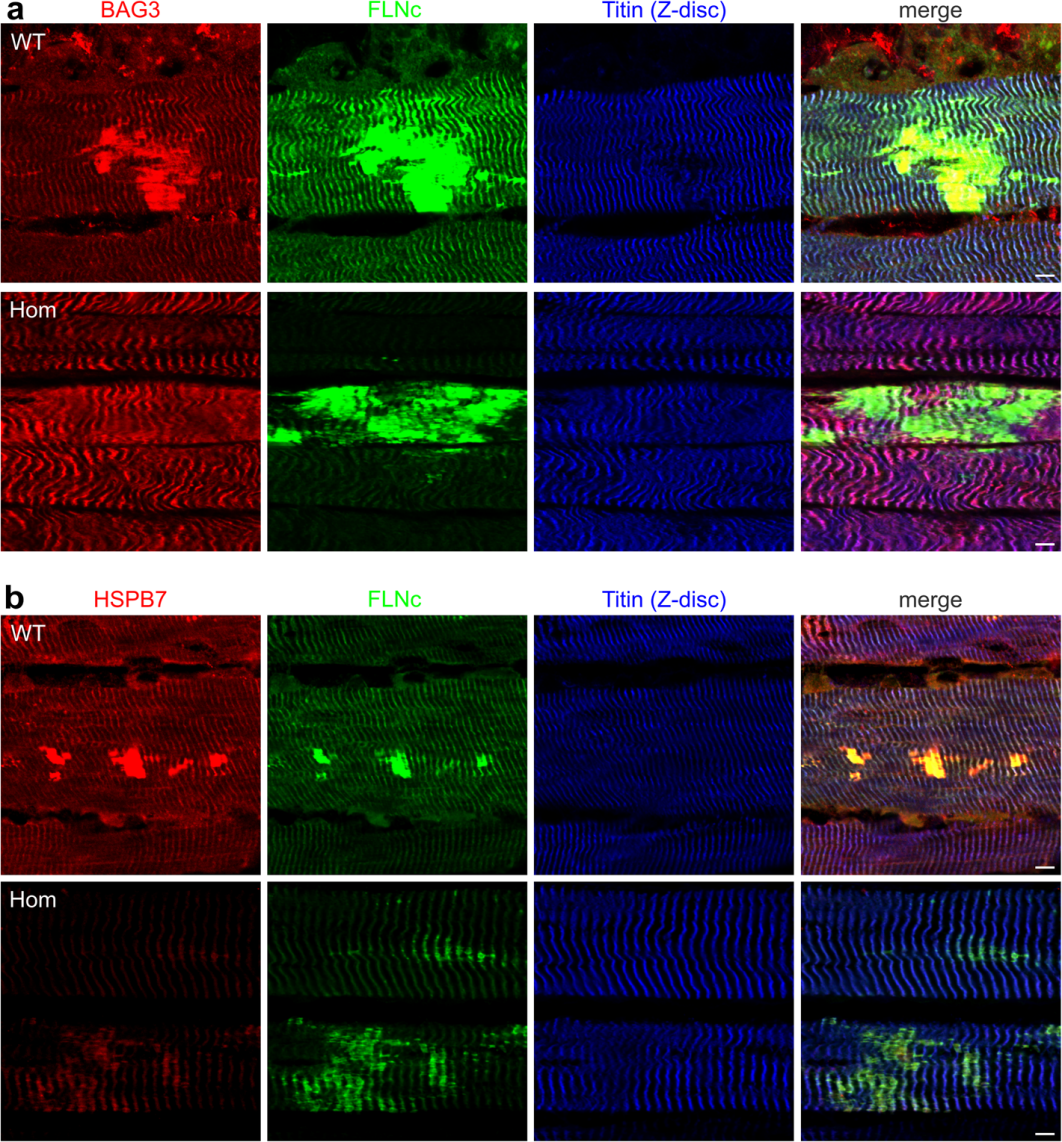
Immunolocalization of BAG3 and HSPB7 in muscle fibers of adult wildtype and Hom animals. **a** In WT fibers BAG3 colocalizes together with FLNc in myofibrillar lesions and Z-discs identified with an antibody against an epitope of titin close to the Z-disc. In Hom muscle fibers, BAG3 localizes to Z-discs, but not to lesions. **b** In WT fibers also HSPB7 colocalizes with FLNc in myofibrillar lesions and Z-discs. In Hom muscle fibers, the small amount of HSPB7 that is expressed mainly localizes to lesions. Note that in all cases, identical scan settings were applied. For all experiments the RR90 antibody was used to detect FLNc. Bars: 5 μm

Pathways involved in elimination of misfolded or damaged proteins by autophagy involve BAG3 and HSPB1, HSPB5 and HSPB8, whereas HSPB7 functions independently from BAG3. We analyzed which of these proteins are associated with myofibrillar lesions in WT and Hom muscle fibers. Antibodies against BAG3 as well as HSPB7, brightly stained lesions and Z-discs in muscle fibers from WT mice (Fig. 7a, b, upper panels). By contrast, in the muscle fibers from Hom animals, BAG3 only localized to Z-discs and was not detected in lesions (Fig. 7a, lower panel). The small amounts of HSPB7 that were still expressed in Hom muscle fibers mainly localized to lesions (Fig. 7b, center panel).

### Identification of a putative alternative dimerization site in filamin C

**Fig. 8:**
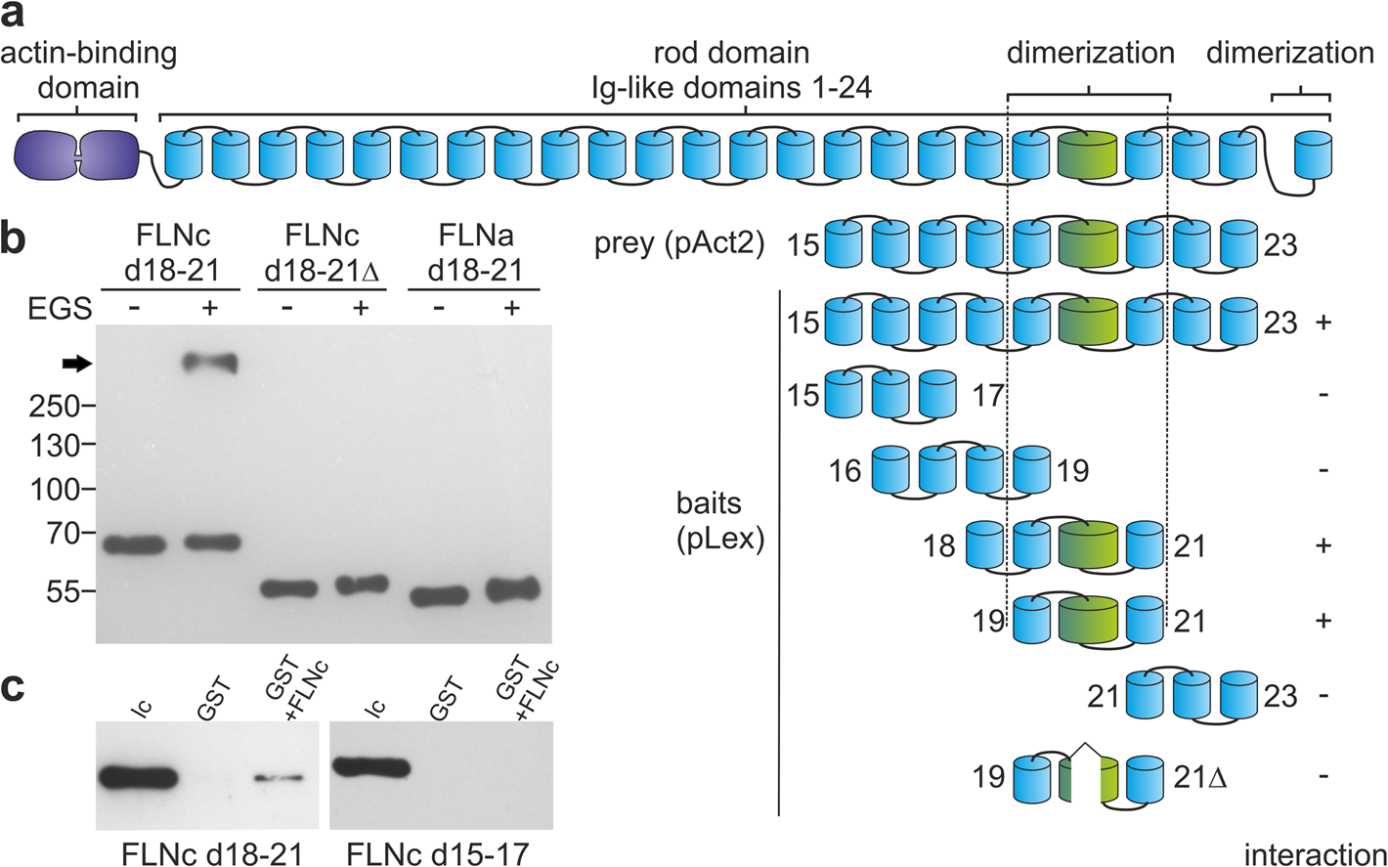
Analysis of a putative second dimerization region in FLNc. **a** Yeast-two-hybrid experiments investigating interaction of FLNc Ig-like domains 15–23 (prey) with different fragments of FLNc as baits. Interaction was found with d15–23, d18–21 and d19–21 of FLNc. Note that deletion of the FLNc specific insertion in Ig-like domain 20 (d19–21Δ) abolished the interaction. +: interaction; −: no interaction. **b** FLNc d18–21 with and without (Δ) insertion in d20, and FLNa d18–21 were incubated in the presence of the cross linker EGS. Separation by PAGE and western blotting with an antibody against the immunotag of the recombinant fragments indicated oligomerization of FLNc d18–21 (arrow) but not of the other constructs. **c** GST pull down assay indicating that GST-FLNc d19–21 (GST + FLNc) binds T7-tagged FLNc d19–21, but not T7-tagged FLNc d15–17. GST alone does not bind any of the FLNc fragments. lc: loading control showing the correct size of the FLNc fragments

Our previous work demonstrated that the *FLNC* p.W2710X mutation abolishes the dimerization property of its Ig-like domain 24 [47, 86]. Indications for the presence of an alternative dimerization site within the rod 2 domain of FLNb [70] prompted us to investigate whether also FLNc might have a second dimerization site. We therefore tested the interaction of a construct encompassing FLNc Ig-like domains 15–23 with several other fragments in the yeast-two-hybrid system. Indeed, several fragments interacted (d15–23, d18–21, d19–21), whereas several others (d15–17, d16–19, d21–23) did not. This identified Ig-like domains 19–21 as the smallest interacting fragment. Notably, deletion of the FLNc-specific insertion in Ig-like domain 20 abolished this interaction (Fig. 8a). To confirm this interaction biochemically, we performed cross-linking assays with FLNc Ig-like domains 18–21, similar to those used to show dimerization of Ig-like domain 24 [29, 47]. This resulted in higher molecular mass complexes compatible with oligomerization of this FLNc fragment. As noticed in yeast-two-hybrid assays, deletion of the insertion in Ig-like d20 prohibited the interaction. By contrast, a comparable construct comprising FLNa d18–21 did not seem to interact with itself in this assay (Fig. 8b). To confirm this interaction, we also performed pull-down assays in which a GST-FLNc d18–21 fusion protein bound to GSH-beads interacted with FLNc d18–21, but not with FLNc d15–17 (Fig. 8c). This indicates that FLNc lacking Ig-like domain 24, and probably also the p.W2710X mutant variant, might still be able to dimerize or oligomerize.

## Discussion

Until now more than 200 dominant human mutations in the *FLNC* gene have been identified to be associated with a great variety of myopathies and cardiomyopathies [83]. In addition, very recently a first case of a homozygous, recessive *FLNC* mutation (NM_001458.4:c.1325C > G; p.P442R) was reported to cause a severe congenital myopathy associated with degenerated myofibrils, advanced sarcomeric lesions and protein aggregates containing FLNc, Xin and XIRP2 in patient muscle fibers [40]. In extension to our previous work on human p.W2710X filaminopathies and patient-mimicking heterozygous *Flnc* p.W2711X knock-in mice [11, 36–38, 47, 86], we therefore characterized mice homozygous for this mutation. Our hypothesis was that the sole expression of mutant FLNc, containing a truncated, dimerization-incompetent Ig-like domain 24, would enhance pathological processes, thus allowing more precise analysis of the underlying pathomechanisms. One key finding is that, although general striated muscle development and sarcomere formation are not interfered in these mice, even sedentary animals develop a myopathic phenotype in conjunction with a fulminant sarcomeric lesion pathology. By contrast, mice in which the *Flnc* exons encoding Ig-like domains 21–24 were deleted (Flnc^tm1Lmk^, http://www.informatics.jax.org/allele/key/35197) display neonatal lethality caused by defects in primary myogenesis [14], while inducible cardiac-specific ablation of *Flnc* expression resulted in embryonic death, or death associated with dilated cardiomyopathy in adult mice, respectively [92]. One mechanistic difference explaining the different phenotypes is the fact that the mutations in the latter two mouse models result in highly reduced *Flnc* mRNA expression, most likely caused by nonsense mediated decay (NMD), and consequently hardly or no detectable levels of mutant FLNc protein [14, 92]. Therefore, their lethal phenotype has to be attributed to a complete loss of FLNc function. By contrast, the p.W2711X mutation, residing in the last exon, bypasses NMD, resulting in expression of *Flnc* mRNA at normal levels. This, in turn, implies that the reduced level of mutant protein in our Hom mice is most likely the result of increased FLNc protein degradation. Notably, *FLNC* is a dosage-sensitive gene and reduced expression of FLNc causes myopathies [26], indicating that the highly reduced FLNc protein level of our Hom mice is a major mechanism contributing to their phenotype. In this respect it is interesting to note that mass spectrometry analysis of skeletal muscle fibers of heterozygous p.W2711X mice indicated that FLNc levels are reduced to 85.0% (*p* = 0.035) and 70.1% (*p* = 0.0054) in type I and type II fibers, respectively (ProteomeXchange submission PXD020097). These differences were previously not detected by non-quantitative western blotting [11].

It is difficult to separate the effect of the reduction of FLNc from that of the expression of mutant FLNc in our mice. Surprisingly few data are available on FLNc expression levels in myopathy patients with an FLNc mutation. In a family with a nonsense mutation (p.F1720LfsX63) that leads to NMD of the mutant mRNA and reduced expression of only wildtype FLNc, the phenotype is compatible with distal myopathy without the typical signs of MFM such as protein aggregation [26]. Patients with an in frame deletion mutation in Ig-like domain 7 (p.V930_T933del) express normal or even increased levels of FLNc [69], and expression of this unstable and misfolded protein [38] is associated with the formation of aggregates and lesions [11, 38, 69]. A patient with a homozygous p.P442R mutation in Ig-like domain 2 expressed a normal level of unstable FLNc protein and his muscle fibers contained Xin- and FLNc-positive aggregates [40]. This indicates that low FLNc expression levels cause a myopathic phenotype without or with limited aggregates or lesions, whereas the expression of toxic FLNc protein results in lesion and/or aggregate formation.

FLNc is degraded by multiple pathways, including CASA [1, 74], proteasomal degradation [2], and cleavage mediated by calpain 3 [27] or KY protein [3]. Particularly important components for the detection and elimination of damaged FLNc are small heat-shock proteins (sHSPs). The increased expression level of HSPB1, HSPB5 and HSPB8 (and other autophagy-related proteins) especially in type II fibers of our Hom mice (Fig. 2c) points at enhanced autophagic activity. In accordance, in the muscle fibers of MFM-filaminopathy patients, HSPB1 and HSPB5 are strongly upregulated, translocated away from Z-discs [75] and highly concentrated in pathologic aggregates [39]. Notably, these sHSPs interact and cooperate with the co-chaperone and key CASA component BAG3. A major difference between WT, heterozygous p.W2711X and Hom mice is that only in Hom mice BAG3 is not a component of myofibrillar lesions. This implies that an important mechanism contributing to the pathophysiology of the p.W2711X mutation is a lack of full CASA functionality that would be necessary for sufficient repair of the enhanced lesion formation in our Hom mice.

By contrast, the expression level of HSPB7 is highly reduced in the muscle fibers of Hom mice. HSPB7 interacts with the dimerizing domain 24 of FLNc, but not with the p.W2710X variant [31]. HSPB7 was proposed to facilitate early post-damage processing of FLNc and titin [50] and it functions independently from BAG3 [87]. In mice, the complete lack of HSPB7 leads to a progressive myopathy associated with disarray of myofibrils [31]. Cardiac-specific HSPB7 ablation even results in embryonic lethality, concomitant with abnormal actin filament bundles (AABs) within sarcomeres, ultimately resulting in myofibril disorganization [90]. AABs resemble the microlesions we observed in mouse hearts after aortic constriction and in the skeletal muscle fibers of our p.W2711X mice and MFM patients [11, 34]. These AABs lack tropomyosin, troponin, MyHC and myomesin and are non-contractile [90]. We propose that only in the absence of these proteins, actin cross-linking proteins like FLNc and Xin [57] can bind and stabilize these actin filament bundles. The extremely low expression level of HSPB7 in Hom mice and its inability to interact with mutant FLNc is therefore implied as a major disease mechanism aggravating myofibrillar instability and resulting in increased abundance of micro- and macrolesions. Accordingly, interference with HSPB7 expression in zebrafish and human cardiomyocytes also results in increased load by damaged proteins and stimulation of autophagic pathways [50]. Further studies are necessary to resolve the cause for its strongly reduced expression in our Hom mice, and to elucidate its precise role, both in actin-based assembly and regulation of autophagic pathways [13].

In p.W2711X FLNc only Ig-like domain 24 is misfolded, whereas the other 23 domains remain intact and functional. It is therefore reasonable to assume that most of the functionality of the WT protein is retained, which might explain the still relatively subtle phenotype. Heterozygous p.W2711X knock-in mice, which express a mixture of WT and mutant FLNc, revealed an even milder clinical phenotype, with strain-induced sarcomeric macro- and microlesions as most prominent finding [11]. This suggests that mutant FLNc interferes with the maintenance of structural integrity of sarcomeric Z-discs and mechanical stress resistance. Our present work shows that the sole and reduced expression of mutant FLNc significantly aggravates this phenotype. Human p.W2710X FLNc is prone to aggregate formation in vivo and in vitro [47, 86], and its heterozygous expression results in myopathy with lesion and aggregate formation in muscle fibers around the age of 45 years [36, 86]. The reason why we did not find protein aggregates even in aged Hom mice may simply be that mice do not reach a sufficient age to develop comparable aggregates. We already previously postulated that lesions most likely define preclinical disease stages, preceding the formation of protein aggregates [11]. It was shown that eccentric exercise [21, 53] and electrical pulse stimulation of cultured myotubes [55] leads to increased lesion formation. This would imply that particularly muscles exposed to above average mechanical stress should be particularly affected. In mice, the soleus muscle is the most biomechanically strained muscle during walking, i.e. already under standard housing conditions [30]. Interestingly, FLNc shows its highest expression level in the soleus muscle (Fig. 1a), and here we observed a higher incidence of lesions in comparison to other muscles (results not shown). These findings imply that in humans, muscles which are most strongly strained during walking (like the gastrocnemius, semimembranosus and rectus femoris muscles), should be most strongly affected [30]. Indeed, muscle imaging revealed that the gastrocnemius and semimembranosus muscles are among the most severely affected muscles in human filaminopathy patients [38]. It is thus very likely that strong physical exercise would further aggravate this pathology, an issue important for patient management and to be addressed in future experiments.

Filamins can act as sensors for mechanical stress [17, 41, 62, 63, 74]. FLNc is a highly dynamic protein capable of relocating within seconds, e.g. upon inflicting muscle damage by laser-induced microdamage or electrical pacing stimulation [43, 51, 55]. In line with this, in a large variety of human muscle diseases FLNc is dramatically redistributed away from the Z-disc and recruited to subcellular areas with functional imbalances and/or particular mechanical strain such as protein aggregates in myofibrillar myopathies, cores in central and minicore myopathies, subsarcolemmal space in dystrophinopathies, and target lesions in neurogenic atrophies [8, 12, 54, 68, 72, 86]. This is substantiated in our Hom mice that show an almost complete relocalization of (the remaining) mutant FLNc from Z-discs to myofibrillar lesions and MTJs (see Figs. 3 and 4, Additional file 1: Figure S4). Notably, this relocalization is not influenced by the lack of the last 16 amino acids. Whereas the complete deletion of Ig-like domain 24 abolishes interaction with sarcoglycans [27], truncation of the C-terminal 16 amino acids alone does not [47]. Likewise, the interaction with Xin, aciculin/PGM5, myotilin, FATZ, myopodin and other ligands that bind FLNc in more amino-terminal regions [20, 45, 51, 81, 82] is not influenced. Accordingly, most of these proteins are found in lesions together with FLNc in the WT as well as in the heterozygous and homozygous mutant situation [19, 34, 43, 51, 55]. Furthermore, in a zebrafish model with knockdown of endogenous FLNc, heterologous expression of p.W2710X human FLNc rescues the severe fiber disintegration phenotype, confirming sufficient functionality of the mutant protein [65]. Since also in these fish mutant FLNc is sequestered to attachment sites of myofibrils (comparable to MTJs) and protein aggregates, the authors concluded that the accompanying lack of FLNc (either WT or mutant) in Z-discs is a major cause of myofibrillar instability. The association of FLNc with Xin actin-binding repeat-containing proteins in MTJs and non-striated parts of myofibrils, including lesions, reduces FLNc dynamics and mobility [43] which might protect the protein in these locations from degradation, and explain its increased localization in these structures.

It is widely accepted that dimerization of filamins is an essential feature of this protein family [15, 52, 71] and it was surprising that p.W2711X Hom mice do not have a more severe phenotype. This might imply that dimerization is not an essential property of FLNc in striated muscles, or that FLNc does not really cross-link actin filaments in myofibrils. Indeed, an FLNc variant completely lacking Ig-like domain 24 exhibited Z-disc binding but, when compared to the full length variant, higher dynamics in fluorescence recovery after photobleaching (FRAP) experiments [61]. One possible explanation could be an indirect FLNc dimerization achieved by refilins, that contain multiple filamin binding sites [24], or other binding partners capable of forming dimers such as myotilin [66, 85]. An alternative explanation would be the presence of an additional oligomerization site within FLNc as reported for FLNb [70], possibly resulting from intermolecular interactions comparable to the intramolecular interactions reported for domain pair 20–21 in FLNa [28, 41]. Indeed, our yeast two hybrid and in vitro interaction studies were indicative of an additional dimer forming region distinct from Ig-like domain 24.

This leads to the ultimate question what the function of FLNc is in sarcomeric lesions. Considering the fact that FLNc has a plethora of binding partners with highly different functions, we assume that its major task is to orchestrate sarcomere repair by acting as an adaptor protein capable of recruiting both signaling and structural components. Our immunostainings of macrolesions imply that locally titin molecules are maximally stretched to a Z-disc epitope spacing of approximately 7.2 μm. This distance is compatible with the length of two titin molecules stretched to their maximal extension [7, 35]. In these regions FLNc is relocated from Z-discs to the space between these far distant titin epitopes (see Fig. 4), where a non-contractile and elastic filamentous scaffold is formed to reduce the mechanical stress in order to prevent further damage and to retain the continuity of the contractile apparatus [43, 55]. As a protein with many binding partners, FLNc can then recruit other proteins necessary for repair of the damaged sarcomeres [43].

Further studies addressing the sequential molecular events and the time course of lesion formation and repair, and the involvement of autophagy in this context are needed to understand this crucial process in detail.

## Conclusions

Homozygosity for the MFM-associated p.W2711X mutation in *Flnc* in mice leads to increased lesion formation in skeletal muscle fibers even without additional exposure to strenuous mechanical stress. Our analyses revealed the following major mechanisms contributing to the pathophysiological condition of filaminopathy:

1. although *Flnc* mRNA expression levels are not reduced, muscle fibers from Hom mice express highly reduced levels of FLNc protein.
2. mutant FLNc relocates from Z-discs to particularly mechanically strained parts of muscle cells;
3. muscle fibers from Hom mice display dysregulated sHSP expression: while HSPB7 is lacking, HSPB1, HSPB5 and HSPB8 are upregulated; the absence of HSPB7 may directly contribute to sarcomere disorganization;
4. dysregulation of CASA is indicated by the absence of BAG3 from lesions and concomitant upregulation of the adapter protein SYNPO2; consequently, clearance of mechanically damaged proteins from lesions is compromised.

Homozygous *FLNC* p.W2711X mice will be an excellent tool for investigations aiming at understanding sarcomeric lesion formation, and to find possibilities to prevent muscle weakness by lesion formation or to find methods to enhance lesion repair.

## Supplementary information

**Additional file 1: Figure S1.** Histochemistry performed on 8-month-old wild type (WT) and mutant (Hom) mouse soleus muscles. **Figure S2.** Analysis of fibre types in soleus muscle of adult and aged wildtype and Hom mice. **Figure S3.** Distribution of filamin C and vinculin in myotendinous junctions. **Table S1.** Specification of primary antibodies used in this study. **Table S2.** Sequence of oligonucleotides used for quantitative real time PCR.

## Data Availability

The datasets used and/or analyzed during the current study available from the corresponding author on reasonable request. Mass spectrometry proteomics data have been deposited to the ProteomeXchange Consortium via the PRIDE partner repository with the dataset identifier PXD020097.

## Abbreviations

BAG3: Bcl2-associated athanogene 3 protein
BSA: Bovine serum albumin
CLB: Cross-link buffer
COX: Cytochrome oxidase
EDL: Extensor digitorum longus
EGFP: Enhanced green fluorescent protein
FATZ: Filamin-, actinin-, and telethonin-binding protein of the Z-disc
FDR: False discovery rate
FLN: Filamin
GAPDH: Glyceraldehyde-3-phosphate dehydrogenase
GST: Glutathione S-transferase
HE: Haematoxylin and eosin
HSPB7: Heat shock protein beta-7
Ig-like: immunoglobulin-like
LMD: Laser microdissection
MFM: Myofibrillar myopathy
MHC: Myosin heavy chain
MS: Mass spectrometry
MTJ: Myotendinous junction
NMD: Nonsense mediated decay
PAGE: Polyacrylamide gel electrophoresis
PBS(T): Phosphate-buffered saline (with Tween 20)
PGM: Phosphoglucomutase
PSMs: Peptide spectrum matches
PVDF: Polyvinylidenfluoride
RT-qPCR: Reverse transcription quantitative polymerase chain reaction
SDH: Succinate dehydrogenase
TA: Tibialis anterior
TBS(T): Tris-buffered saline (with Tween 20)
WT: Wildtype
