# Supplementary material for: Homozygous expression of the myofibrillar myopathy-associated p.W2710X filamin C variant reveals major pathomechanisms of sarcomeric lesion formation
Source: Acta Neuropathol Commun. 2020 Sep 4;8:154. doi: 10.1186/s40478-020-01001-9 (PMC7650280; doi:10.1186/s40478-020-01001-9)
Supplement: Supplementary file 1 — Additional file 1: Figure S1. Histochemistry performed on 8-month-old wild type (WT) and mutant (Hom) mouse soleus muscles. Figure S2. Analysis of fibre types in soleus muscle of adult and aged wildtype and Hom mice. Figure S3. Distribution of filamin C and vinculin in myotendinous junctions. Table S1. Specification of primary antibodies used in this study. Table S2. Sequence of oligonucleotides used for quantitative real time PCR. [file 40478_2020_1001_MOESM1_ESM.doc]

**Additional file 1**

**Sarcomeric pathology induced by homozygous expression of the myofibrillar myopathy-associated p.W2711X filamin C mutant**

Julia Schuld, Zacharias Orfanos, Frédéric Chevessier, Britta Eggers, Lorena Heil, Julian Uszkoreit, Andreas Unger, Gregor Kirfel, Peter F. M. van der Ven, Katrin Marcus, Wolfgang A. Linke, Christoph S. Clemen, Rolf Schröder, Dieter O. Fürst

Corresponding authors:

Dieter O. Fürst, University of Bonn, Institute for Cell Biology, Department of Molecular Cell Biology, Ulrich-Haberland-Str. 61a, 53121 Bonn, Germany. E-mail: dfuerst@uni-bonn.de; Phone: +49-228-735301; Fax: +49-228-735302

Rolf Schröder, Institute of Neuropathology, University Hospital Erlangen, Friedrich-Alexander University Erlangen-Nürnberg, Schwabachanlage 6, 91054 Erlangen, Germany. E‑mail: rolf.schroeder@uk-erlangen.de; Phone: +49-9131-8534782; Fax: +49-9131-8526033


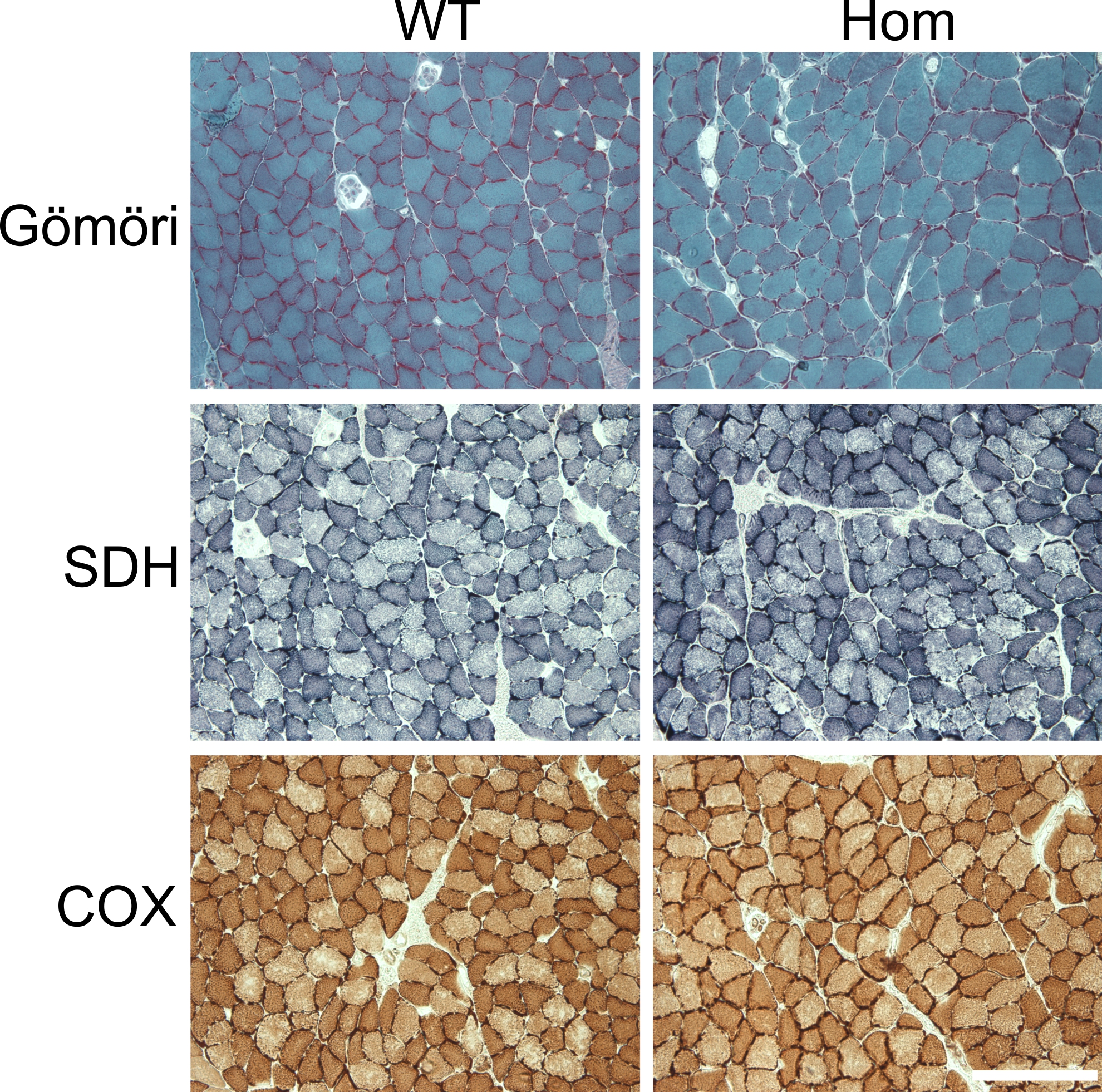


**Figure S1**. Histochemistry performed on 8-month-old wild type (WT) and mutant (Hom) mouse soleus muscles. No remarkable differences can be noted between WT and mutant mice using modified Gömöri trichrome, or the two mitochondrial stains succinate dehydrogenase (SDH) and cytochrome oxidase (COX). Scale bar: 200 μm.


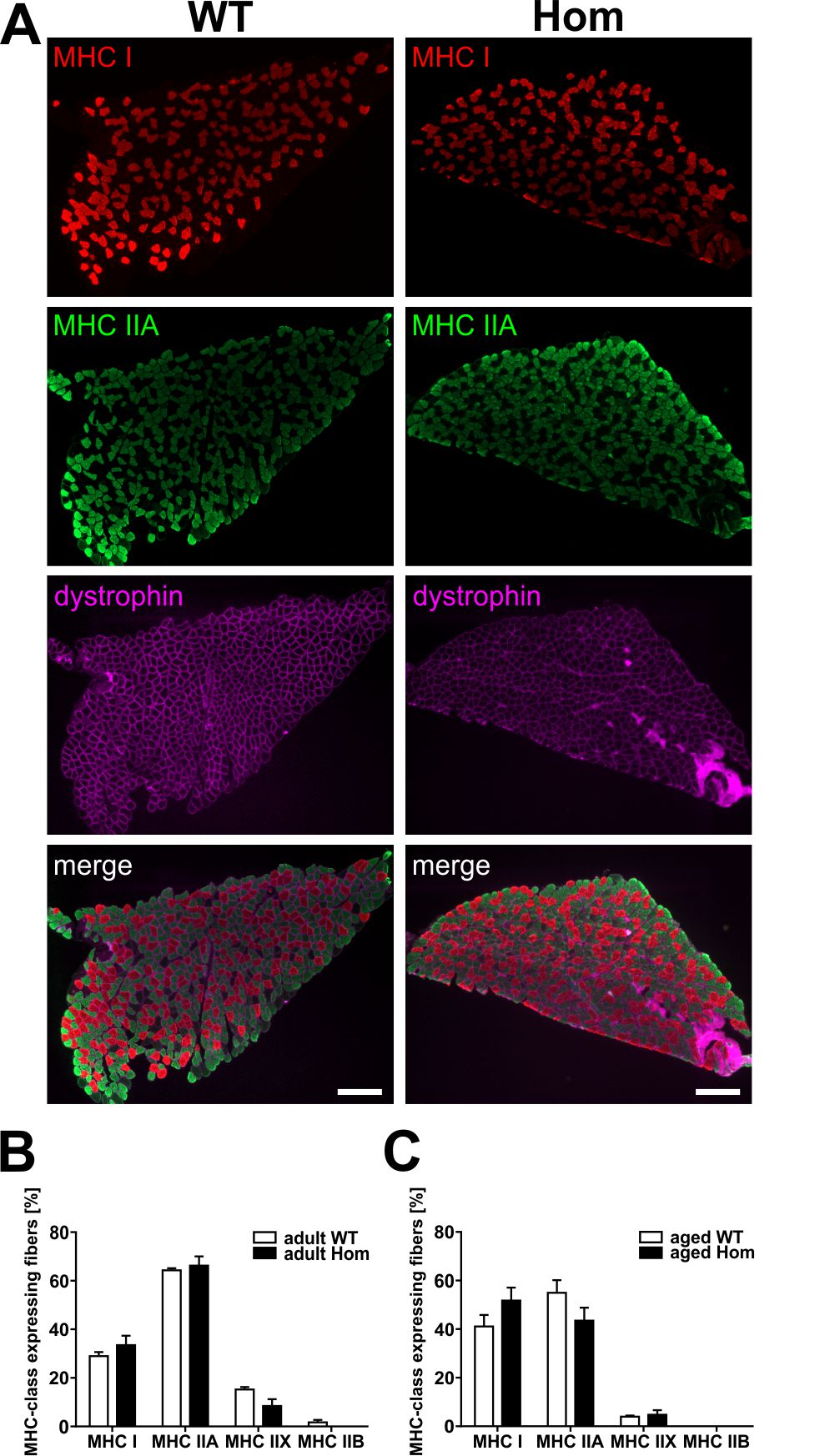


**Figure S2.** Analysis of fiber types in soleus muscle of adult and aged wildtype and Hom mice. A. Immunolocalization of myosin heavy chain type I (MHC I) or type IIA (MHC IIA) and dystrophin (to identify individual fibers) in transverse cryosections of soleus muscle from adult WT or Hom animals (n=3). B,C. The number of fibers expressing a specific myosin heavy chain isoform does not differ significantly between adult and aged WT and Hom mice.


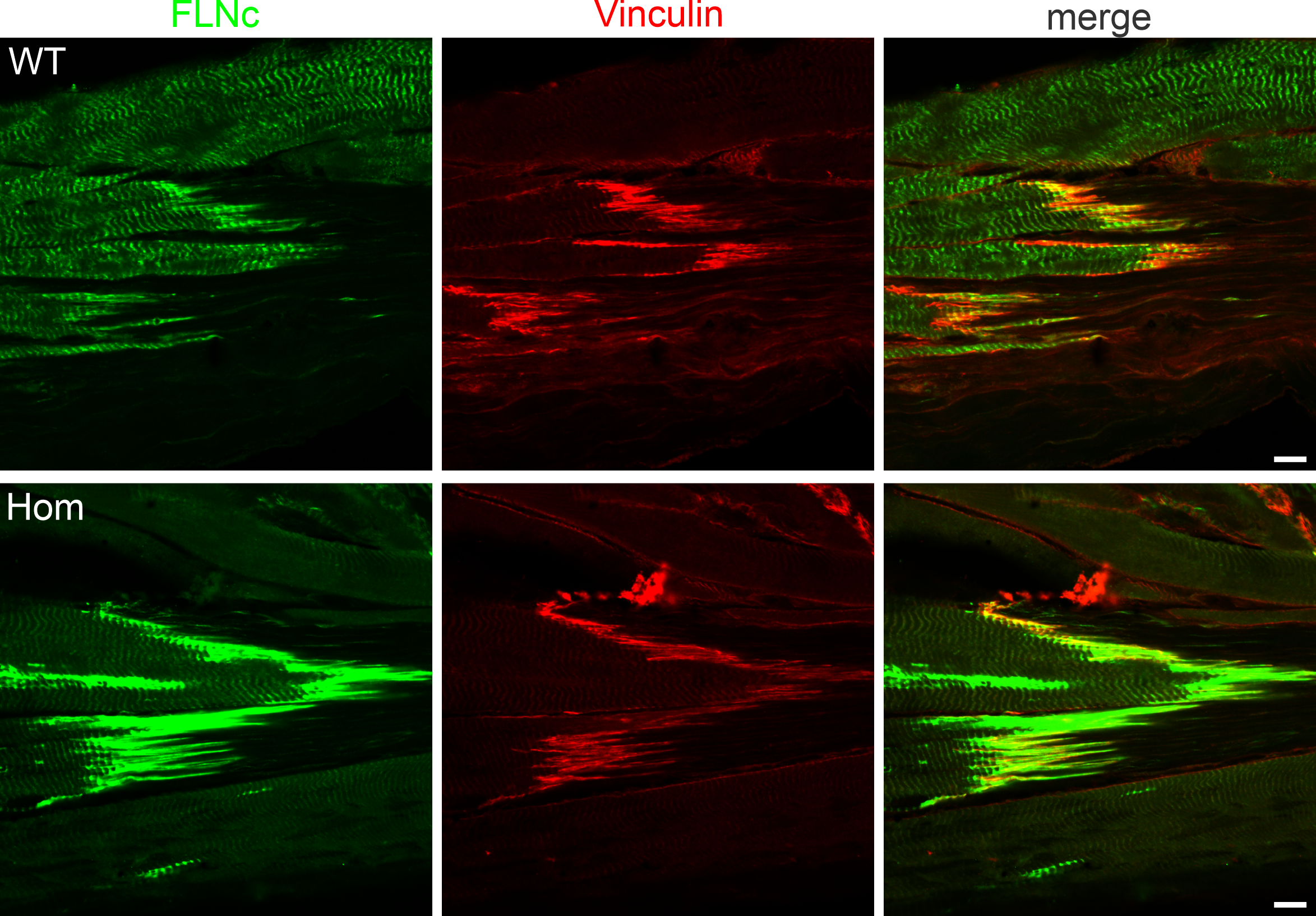


**Figure S3. Distribution of filamin C and vinculin in myotendinous junctions.** Longitudinal cryosections of soleus muscle from a WT and a Hom mouse stained for FLNc and vinculin. Scan settings were identical. Note the accumulation of mutant FLNc within the myotendinous junctions identified by vinculin staining, and the reduced level of FLNc in Z‑discs in Hom mice. Bars: 10 μm.

**Table S1**. Specification of primary antibodies used in this study. Monoclonal antibodies marked "DSHB" were obtained from the Developmental Studies Hybridoma Bank, created by the NICHD of the NIH and maintained at the University of Iowa, Department of Biology, Iowa City, IA 52242.

| **Antigen (Clone)** | **Source/reference** | **Species** | **Immunostaining** | **Immunoblotting** |
| --- | --- | --- | --- | --- |
| α-actinin | (van der Ven et al., 2000) | rabbit polyclonal | 1:50 | 1:500 |
| BAG3 | Proteintech  10599-1-AP | rabbit polyclonal | - | 1:1,000 |
| Desmin | Progen 10570 | rabbit polyclonal | 1:200 | 1:500 |
| Dystrophin  (Mandra1) | DSHB | mouse IgG1 | 1:10 | - |
| Filamin A/C (RR90) | (van der Ven et al., 2000) | mouse IgA | 1:50 | - |
| Filamin C d16-20 | (Kley et al., 2012) | rabbit polyclonal | 1:50 | 1:5,000 |
| Filamin C WT | (Chevessier et al., 2015) | rabbit polyclonal | - | 1:100 |
| GAPDH (6C5) | Calbiochem CB1001 | mouse IgG1 | - | 1:1,000 |
| HSPB7 | Proteintech  15700-1-AP | rabbit polyclonal | 1:50 | 1:500 |
| Myomesin  (BB78) | (Vinkemeier et al., 1993) | mouse IgG2a | 1:50 | 1:500 |
| MHC I  (BA-F8) | DSHB | mouse IgG2b | 1:10 | - |
| MHC IIA (SC71) | DSHB | mouse IgG1 | 1:10 | 1:50 |
| MHC IIX  (6H1) | DSHB | mouse IgM | 1:10 | - |
| MHC IIB  (BF-F3) | DSHB | mouse IgM | 1:10 | - |
| MHCS  (NCL-MHCs) | Novocastra,/Leica Microsystems | Mouse IgG1 | 1:40 | - |
| Titin Z-disc (T12) | (Fürst et al., 1988) | mouse IgG1 | - | 1:200 |
| α-Tubulin (YL1/2) | (Wehland et al., 1983) | rat IgG2a | - | 1:500 |
| Vinculin  (hVin-1) | Sigma Aldrich/Merck  V9131 | mouse IgG1 | 1:100 | - |
| Xin (XR1) | (van der Ven et al., 2006) | mouse IgG1 | 1:5 | 1:50 |

**Table S2**. Sequence of oligonucleotides used for quantitative real time PCR

| *Gene* | Forward primer | Reverse primer |
| --- | --- | --- |
| *B2m* | AATGTGAGGCGGGTGGAACTG | CATGGCTCGCTCGGTGACC |
| *Gapdh* | AGGTCGGTGTGAACGGATTTG | TGTAGACCATGTAGTTGAGGTCA |
| *Flna* | AAGCCCTCTGCAGTTCTATGTTGAT | GCAAACGTTTCAGCAGACAGGGTT |
| *Flnb* | CTGAGGAGATTATTCACCCGGA | GCGCTGATGGTATCTACCGTG |
| *Flnc* | CCTTACTCGCCCTTCCGCATCCAT | CTCGGAGCTGTGTAGTAGATGTC |

**References**

Chevessier, F., Schuld, J., Orfanos, Z., Plank, A.-C., Wolf, L., Maerkens, A., Unger, A., Schlötzer-Schrehardt, U., Kley, R.A., von Hörsten, S., et al. (2015). Myofibrillar instability exacerbated by acute exercise in filaminopathy. Hum. Mol. Genet., 7207-7220.

Fürst, D.O., Osborn, M., Nave, R., and Weber, K. (1988). The organization of titin filaments in the half-sarcomere revealed by monoclonal antibodies in immunoelectron microscopy: a map of ten nonrepetitive epitopes starting at the Z line extends close to the M line. J. Cell Biol. *106*, 1563-1572.

Kley, R.A., Serdaroglu-Oflazer, P., Leber, Y., Odgerel, Z., van der Ven, P.F.M., Olivé, M., Ferrer, I., Onipe, A., Mihaylov, M., Bilbao, J.M., et al. (2012). Pathophysiology of protein aggregation and extended phenotyping in filaminopathy. Brain *135*, 2642-2660.

UniProt Consortium, T. (2018). UniProt. The universal protein knowledgebase. Nucleic Acids Res. *46*, 2699.

van der Ven, P.F.M., Ehler, E., Vakeel, P., Eulitz, S., Schenk, J.A., Milting, H., Micheel, B., and Fürst, D.O. (2006). Unusual splicing events result in distinct Xin isoforms that associate differentially with filamin C and Mena/VASP. Exp. Cell Res. *312*, 2154-2167.

van der Ven, P.F.M., Obermann, W.M.J., Lemke, B., Gautel, M., Weber, K., and Fürst, D.O. (2000). Characterization of muscle filamin isoforms suggests a possible role of γ-filamin/ABP-L in sarcomeric Z-disc formation. Cell Motil. Cytoskeleton *45*, 149-162.

Vinkemeier, U., Obermann, W., Weber, K., and Fürst, D.O. (1993). The globular head domain of titin extends into the center of the sarcomeric M band. cDNA cloning, epitope mapping and immunoelectron microscopy of two titin-associated proteins. J. Cell Sci. *106*, 319-330.

Wehland, J., Willingham, M.C., and Sandoval, I.V. (1983). A rat monoclonal antibody reacting specifically with the tyrosylated form of alpha-tubulin. I. Biochemical characterization, effects on microtubule polymerization in vitro, and microtubule polymerization and organization in vivo. J. Cell Biol. *97*, 1467-1475.
